# Regulation of hyphal development by protein kinase A, stress-responsive MAP kinases, and calcineurin via transcription factors Sfl1 and Sfl2 in *Candida albicans*

**DOI:** 10.1128/msphere.00689-25

**Published:** 2026-01-08

**Authors:** Misty R. Peterson, Shannon Au, Andrew Nhat Ho, Haoping Liu

**Affiliations:** 1 Department of Biological Chemistry, School of Medicine, University of California Irvine 8788, Irvine, California, USA; CNRS-Inserm-Université Côte d'Azur, Nice, France

**Keywords:** transcription factors (Sfl1, Sfl2), protein kinase A, MAP kinases, calcineurin, hyphal development, *Candida albicans*

## Abstract

*Candida albicans* is a major human fungal pathogen whose ability to undergo reversible morphological transitions between yeast and hyphal growth forms represents a key virulence trait. While the cAMP-protein kinase A (PKA) pathway is essential for initiating hyphal growth *in vitro*, it is dispensable for filamentation *in vivo*, yet the molecular mechanisms underlying PKA-dependent and -independent hyphal development remain incompletely understood. Sfl1 and Sfl2 are homologous heat shock transcription factors that antagonistically regulate hyphal development, with Sfl1 repressing and Sfl2 promoting filamentation. Here, we use site-specific mutagenesis to dissect how PKA, stress-responsive MAP kinases, and the phosphatase calcineurin regulate Sfl1 and Sfl2 function. Serine-to-alanine (S-to-A) substitutions at predicted PKA phosphorylation sites activated both factors, while serine-to-aspartate (S-to-D) mutations inhibited their activity. *SFL1^PKA A^* cells suppressed hyphal initiation and failed to downregulate *NRG1*, a key repressor of hyphal development. Genetic inactivation of Sfl1 bypassed Tpk2 requirements; however, S-to-A substitutions at the predicted PKA sites in the hyphal regulator Efg1 blocked hyphal initiation regardless of Sfl1 status. *SFL2^PKA DD^* reduced hyphal formation while *SFL2^PKA AA^* enhanced filamentation compared to wild-type *SFL2*. Environmental stresses regulate these factors through distinct post-translational mechanisms: phosphomimetic mutations at MAPK sites destabilized Sfl1 and promoted hyphal initiation even in *SFL1^PKA A^* cells, whereas Sfl2 lacks equivalent MAPK sites but contains calcineurin-binding motifs critical for filamentation under salt stress. This study reveals how Sfl1 and Sfl2 integrate nutritional and stress signals to control hyphal morphogenesis through both PKA-dependent and -independent regulatory mechanisms.

**IMPORTANCE:**

*Candida albicans* exists as a commensal yeast in healthy individuals but becomes an invasive pathogen when host immunity is compromised. Its ability to switch between yeast and hyphal forms is crucial for pathogenesis. While the cAMP-protein kinase A (PKA) pathway is essential for hyphal induction *in vitro*, filamentation occurs independently of PKA during host infection. This study elucidates how the transcriptional regulators Sfl1 and Sfl2 integrate nutritional and stress signals to control morphological transitions. Through site-specific mutagenesis of conserved target sites for protein kinase A, stress-responsive MAP kinases, and the phosphatase calcineurin in Sfl1 and Sfl2, we demonstrate their roles in orchestrating hyphal development. These findings advance our understanding of how *C. albicans* modulates its morphology in response to host conditions, providing mechanistic insights into the regulatory networks important for both commensal colonization and invasion.

## INTRODUCTION

*Candida albicans* is a prevalent fungal pathogen in humans (1). Disseminated candidiasis has an estimated mortality rate of 40%, even with antifungal drug treatment (2, 3). A key virulence trait of *C. albicans* is its ability to switch between yeast, pseudohyphal, and hyphal growth forms (4). This morphological flexibility is regulated by a complex network of transcription factors that control gene expression, morphology, and virulence (5).

Hyphal development is controlled by two temporally linked regulatory mechanisms: initiation and maintenance of the hyphal transcriptional program (6). Hyphal initiation requires the cAMP-protein kinase A (PKA) pathway and the transcription factors Efg1 and Flo8, which are necessary for a rapid but temporary transcriptional downregulation of the hyphal morphogenesis repressor Nrg1 (6). Hyphal maintenance requires active sensing of the growth environment (6–9). Despite the significance of the cAMP-PKA for virulence and *in vitro* hyphal initiation (10–12), a recent report suggests that *in vivo* hyphal development does not require PKA (13). The molecular mechanisms underlying PKA-dependent and -independent transcriptional regulation of hyphal formation remain incompletely understood.

Sfl1 and Sfl2 are two homologous heat shock transcription factors that antagonistically regulate hyphal development and are both required for full virulence (14–19). Genome-wide location and expression analyses have shown that Sfl1 and Sfl2 directly modulate the expression of key transcriptional regulators of *C. albicans* morphogenesis (15). Sfl1 directly represses the expression of positive regulators of hyphal growth while upregulating *NRG1*(15). Conversely, Sfl2 directly activates the expression of positive regulators of hyphal growth and hyphal-specific genes (15). Although Sfl2 is not required for virulence in systemic candidiasis, it is important for *Candida* dissemination from the gut (16, 19). Despite the central functions of Sfl1 and Sfl2 in hyphal development, upstream signaling pathways controlling their transcriptional activity in *C. albicans* remain undefined.

We previously linked Sfl1 function in hyphal development to stress-responsive kinases in *C. albicans* (20). Consistent with the *sfl1* mutant phenotype, deletions of the core stress response mitogen-activated protein (MAP) kinase Hog1 or the calcium/calmodulin-dependent kinase Cmk1 permits hyphal initiation in acidic media (20). In contrast to the *hog1* mutant phenotype, the deletion of the MAP kinase Cek1 impairs hyphal development (21, 22), highlighting distinct regulatory roles among MAP Kinases. Calcium ions (Ca^2+^) regulate multiple signaling pathways, including the calcineurin pathway, which contributes to hyphal extension (23, 24) and mediates contact sensing (25, 26). Although the individual contributions of PKA, MAPK, and calcineurin pathways to hyphal development are well established, their downstream transcription factor targets remain incompletely understood. Here, by mutating predicted phosphorylation and binding sites in Sfl1/Sfl2, we reveal convergent regulation of these transcription factors by multiple signaling pathways, thereby elucidating a key molecular mechanism controlling hyphal development.

## RESULTS

### Mutations at the predicted PKA site in CaSfl1 regulate hyphal initiation

**Fig 1:**
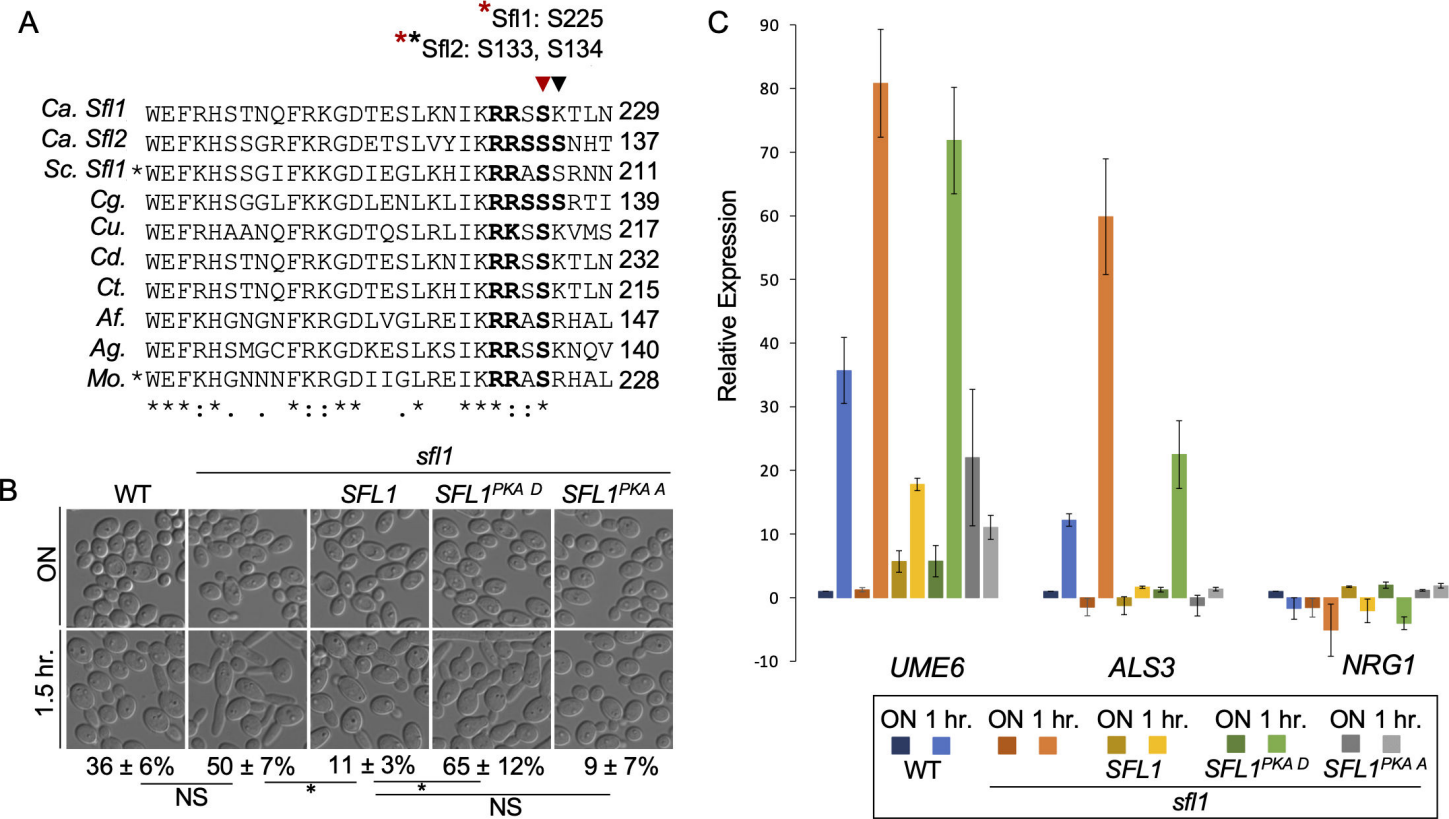
Mutations at predicted PKA site in CaSfl1 regulate hyphal initiation. (**A**) Multiple sequence alignment of segments from Sfl1 and Sfl2 of *C. albicans* (*Ca*) with fungal homologues from *Saccharomyces cerevisiae* (*Sc*) (YOR140W), *Candida glabrata* (*Cg*) (CAGL0107183g)*, Candida auris* (*Cu*) (B9J08_000661), *Candida dubliniensis* (*Cd*) (Cd36_31370), *Candida tropicalis* (*Ct*) (CTRG_00655), *Aspergillus fumigatus* (*Af*) (XP_001481550.1), *Ashbya gossypii* (*Ag*) (NP_985683.1), and *Magnaporthe oryzae* (*Mo*) (MGG_06971). The conserved PKA phosphorylation motif domain is in bold. An asterisk indicates organisms with characterized mutations at the conserved PKA sites (31, 32). (**B**) Hyphal initiation of WT (SN250), *sfl1*, and the *sfl1* transformed with *SFL1* or *SFL1* with PKA mutations is visualized by representative DIC images of cells from overnight culture (YPD, 30°C) and cells inoculated (1:50) into YPD pH 4 at 37°C for 1.5 h. DIC images are representative of three independent experiments and percentages of hyphal initiation are mean ± SD of 300 cells in biological triplicates. Significant differences were calculated by unpaired *t*-test and denoted by an asterisk. *P < 0.01; NS, *P* > 0.05. (**C**) Cells from 1B are also used for gene expression analysis. Gene expression is relative to *CDC28*. Normalization is made to 1 h wild type, which is set to 1, for *UME6* and *ALS3;* and to overnight wild type (set to 1) for *NRG1*. Expression fold change values <1 are depicted as a negative fold change (−1/*x*). The data are from four biological replicates each with three technical replicates for wild type, *sfl1* or *sfl1 ADH1p-SFL1* and two biological replicates for *sfl1 ADH1p-SFL1^PKA D^* or *sfl1 ADH1p-SFL1^PKA A^*.

PKA phosphorylation of *S. cerevisiae* Sfl1 promotes its dissociation from promoter DNA (27, 28). Fungal Sfl1 proteins contain a conserved predicted PKA phosphorylation site (Fig. 1A), located at the carboxyl terminus of the DNA-binding domain (DBD) (29)(Fig. S2). This conserved PKA phosphorylation site is required for filamentation in the rice blast fungus *Magnaporthe oryzae* and *S. cerevisiae* (30, 31). To determine whether this predicted PKA phosphorylation site plays a similar role in *C. albicans,* we generated *SFL1^S225D^* (phosphomimetic, PKA D) and *SFL1^S225A^* (non-phosphorylatable, PKA A) mutations, placed them under the control of the *ADH1* promoter, and transformed the constructs into an *sfl1* deletion mutant.

We used acidic pH for the hyphal initiation assay as the *sfl1* mutant undergoes hyphal initiation under acidic conditions (18, 20). When inoculated from a saturated overnight culture into YPD pH 4 at 37°C, *SFL1*-overexpressing cells exhibited defective hyphal initiation (Fig. 1B). Like wild-type *SFL1*, *SFL1^PKA A^* suppressed hyphal initiation, whereas *SFL1^PKA D^* did not (Fig. 1B). Therefore, the S225D substitution at the predicted PKA phosphorylation site abolished Sfl1’s function as a repressor of hyphal initiation.

Next, we analyzed the effects of these *SFL1* mutations on the hyphal transcription program. We selected *ALS3* and *UME6* as hypha-specific genes (33–36) and *NRG1,* which is downregulated by the cAMP-PKA pathway during hyphal initiation (6, 37). Additionally, *NRG1* and *UME6* are direct transcriptional targets of Sfl1 (15). We analyzed RNA samples from a saturated overnight culture and from cells after 1.5 h of growth in YPD pH 4 at 37°C. In all strains except *SFL1^PKA A^*-expressing strain, *UME6* and *ALS3* expression was higher after 1.5 h in YPD pH 4 at 37°C compared to that of overnight cultures (Fig. 1C). *SFL1^PKA A^* expressing cells failed to induce expression of *UME6* and *ALS3,* and also failed to downregulate *NRG1* expression under hyphal-induction conditions (Fig. 1C). Conversely, *SFL1^PKA D^* expressing cells showed higher *UME6* and *ALS3* expression at 1.5 h compared to that of *SFL1*-expressing cells (Fig. 1C). Thus, Sfl1^PKA D^ is inactive as a repressor, whereas the S225A substitution enhances Sfl1’s repressor activity, consistent with findings in *S. cerevisiae* demonstrating that PKA phosphorylation of Sfl1 promotes its dissociation from promoter DNA (27).

Our genetic evidence with site-specific mutations indicates that PKA phosphorylates CaSfl1 at S225. However, a recent multi-omic study of PKA phosphorylation during hyphal morphogenesis did not detect this modification (38). This observation parallels findings in *S. cerevisiae*, where genetic and biochemical evidence supports PKA regulation of Sfl1 through phosphorylation at conserved sites S207 and S208 (27, 28, 31), yet most phosphoproteomic studies have failed to detect phosphorylation at these sites (39), with only one exception (40). These results suggest that Sfl1 phosphorylation at predicted PKA sites is likely transient and highly dependent on specific cellular conditions.

### *SFL1* deletion bypasses Tpk2 requirement for hyphal initiation

**Fig 2:**
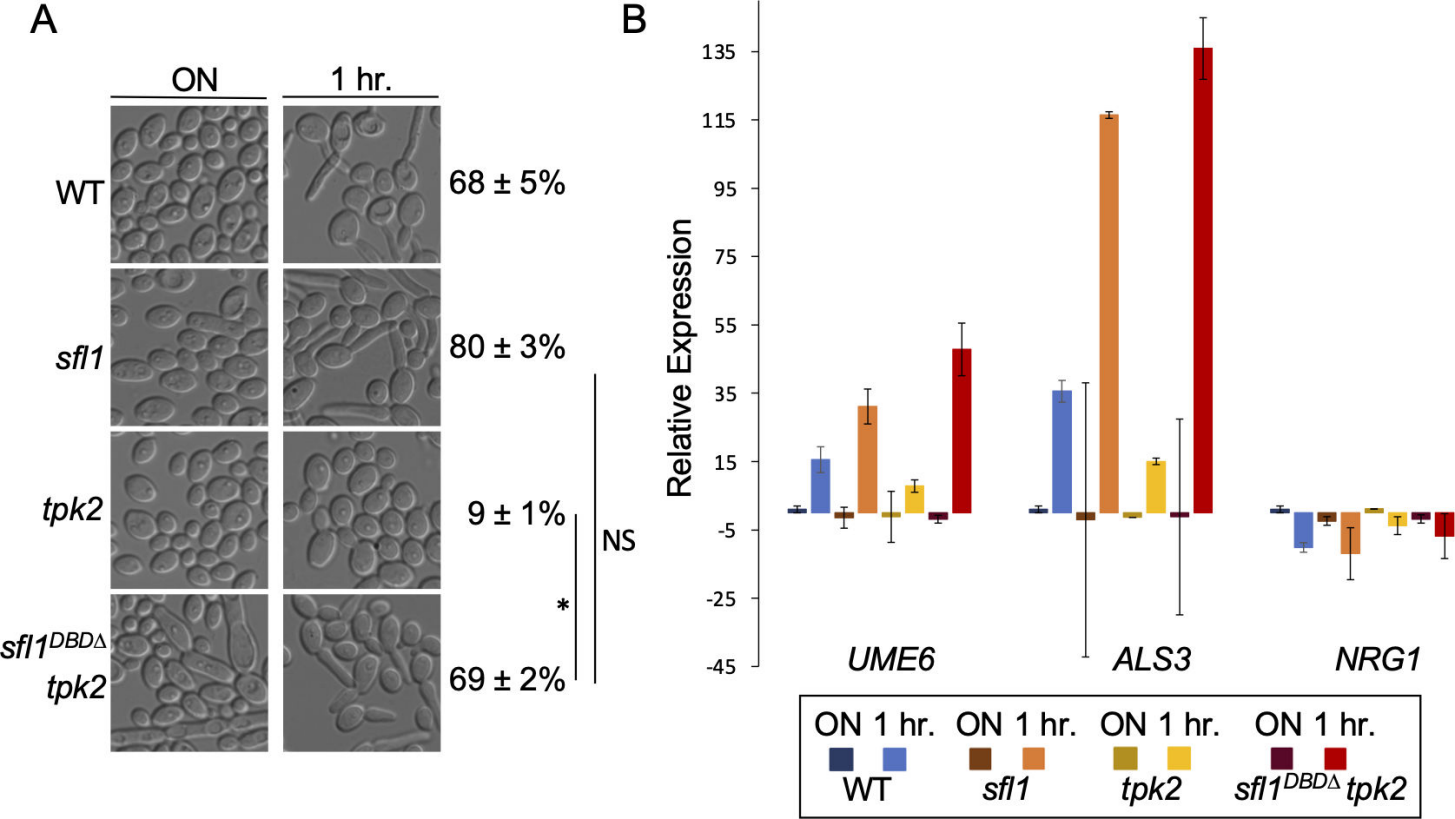
*SFL1*deletion bypasses Tpk2 requirement for hyphal initiation. (**A**) Hyphal initiation of overnight cultures by 1:50 dilution into YPD at 37°C for 1 h. WT*, sfl1*, *tpk2,* and *tpk2 sfl1^DBD∆^* mutants are used. DIC images are representative of three independent experiments, and percentages of hyphal initiation are mean ± SD of 300 cells from triplicate experiments. Significant differences were calculated by unpaired *t*-test and denoted by an asterisk. *P < 0.01; NS, *P* > 0.05. (**B**) Cells from 2A are also used for gene expression analysis as described in Fig. 1C. The data are from two biological replicates each with two technical replicates.

To assess the role of Sfl1 as a downstream effector of PKA signaling in regulating hyphal initiation, we generated an *sfl1^DBD∆^ tpk2* double mutant via CRISPR-Cas9-mediated gene editing (41). While the *tpk2* mutant failed to form hyphae in YPD (pH 6.8) at 37°C, a condition previously used to analyze *tpk2* (8), the *sfl1^DBD∆^ tpk2* double mutant demonstrated robust hyphal induction (Fig. 2A). Transcriptional analysis revealed that *UME6* and *ALS3* expression in the double mutant was higher than that observed in the *tpk2* mutant and comparable to that of the *sfl ^DBD∆^* single mutant (Fig. 2B). These findings suggest that Sfl1 functions as a key downstream target of Tpk2 in regulating hyphal initiation. However, the incomplete restoration of the *sfl1^DBD∆^* mutant filamentation phenotype in the *sfl1^DBD∆^ tpk2* double mutant (Fig. 2A) indicates that Tpk2 has additional targets that contribute to the complex process of hyphal development.

### Regulation of Efg1 by PKA and its relationship with Sfl1 during hyphal initiation

**Fig 3:**
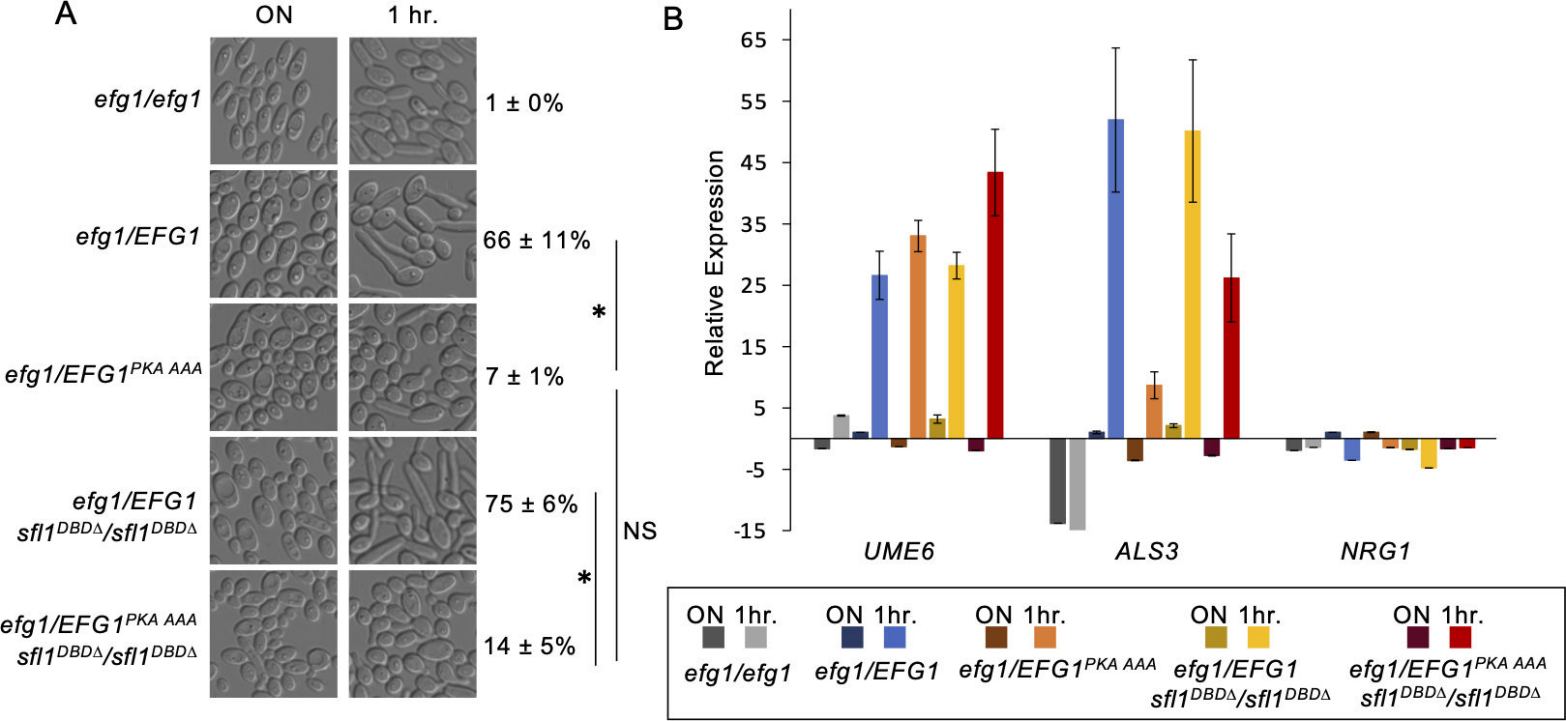
Regulation of Efg1 by PKA and relationship with Sfl1 during hyphal initiation. (**A**) Hyphal initiation of overnight cultures by 1:50 dilution into 37°C YPD for 1 h. Strains used are *efg1/efg1, efg1/EFG1, efg1/EFG1^T206A,T207A, T208A^, efg1/EFG1 sfl1^DBD∆^ /sfl1^DBD∆^,* and *efg1/EFG1^T206A,T207A, T208A^ sfl1^DBD∆^ /sfl1^DBD∆^*. DIC images are representative of three independent experiments, and percentages are means ± SD of 300 cells from triplicate experiments. Significant differences were calculated by unpaired *t*-test and denoted by an asterisk. *P < 0.01; NS, *P* > 0.05. (**B**) Gene expression analysis of *UME6*, *ALS3*, and *NRG1* in cells from 3A, as described in Fig. 1C. The data were from two biological replicates each with two technical replicates.

Efg1 is an essential transcription factor for hyphal initiation and is regulated by Tpk2 (42, 43). Bockmühl et al. demonstrated that alanine substitution of T206, but not of the adjacent T207 or T208, blocked hypha formation on solid media, while a T206E mutation caused hyperfilamentation (42). However, *EFG1^T206A^* showed only partial impairment of hypha formation in liquid media (42). Because Efg1 and Sfl1 interact and function together on promoter DNA (15), we sought to investigate PKA regulation of Efg1 and its relationship with Sfl1 in hyphal initiation. We replaced all three predicted PKA phosphorylation sites with alanine to construct an *EFG1 ^T206A,T207A, T208A^*/*efg1* strain and an *EFG1^T206A,T207A, T208A^*/*efg1 sfl1^DBD∆^/sfl1^DBD^* strain. The *EFG1 ^T206A,T207A, T208A^* mutant showed a significant defect in hyphal initiation when cells from a saturated overnight culture were inoculated into YPD media at 37 °C (Fig. 3A). No significant difference was observed between the *EFG1 ^T206A,T207A, T208A^* single mutant and the *EFG1 ^T206A,T207A, T208A^ sfl1^DBD∆^* double mutant, suggesting that Sfl1 inactivation could not bypass the defect *in EFG1 ^T206A,T207A, T208A^* cells under these experimental conditions (Fig. 3A).

Transcriptional analysis of the selected genes during hyphal initiation for these strains showed that both *EFG1^T206A,^*^T207A,T208A^ and *sfl1^DBD∆^* contributed to changes in their expression (Fig. 3B). *The EFG1^T206A,^*^T207A,T208A^ mutant induced a lower level of *ALS3* but not *UME6* compared to that of the *EFG1* strain during hyphal initiation (Fig. 3B). The control *efg1* mutant showed minimal levels of *ALS3* or *UME6* expression. Reduced *NRG1* expression was observed in the *EFG1* control and the *sfl1^DBD∆^ EFG1* strain, but there was no *NRG1* downregulation in the *efg1* deletion or the *EFG1 ^T206A,T207A, T208A^ sfl1^DBD∆^* double mutant (Fig. 3B). This result suggests that phosphorylation of Efg1 at the predicted PKA sites is essential for the downregulation of *NRG1* expression during hyphal initiation even when Sfl1 is inactivated.

### Phosphomimetic mutations at predicted MAPK sites cause Sfl1 instability and suppress the phenotype of non-phosphorylatable mutation at the predicted PKA site of Sfl1

**Fig 4:**
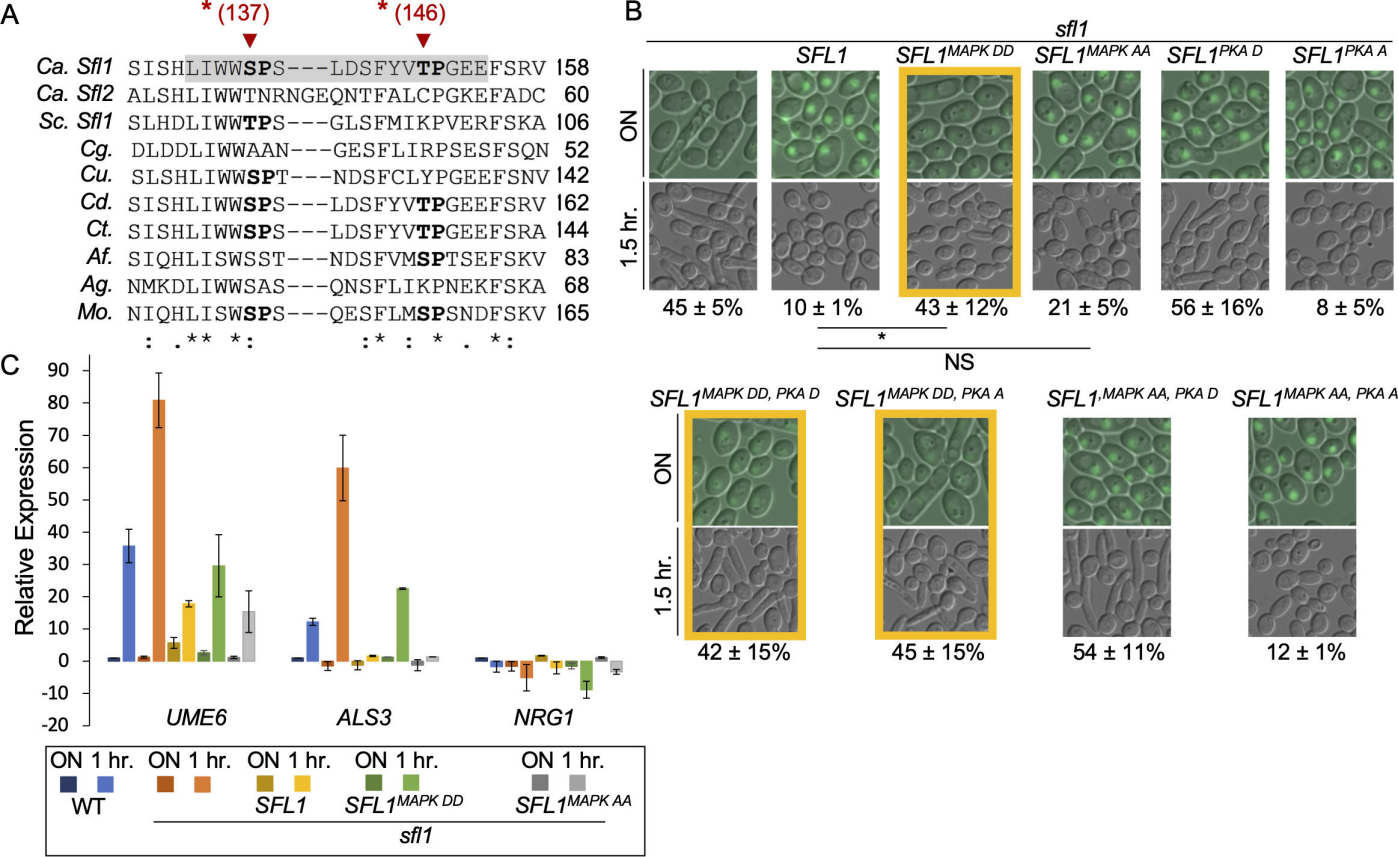
Mutations at predicted MAPK sites in the DNA-binding domain of Sfl1 regulate hyphal initiation in parallel to PKA site regulation. (**A**) Multiple sequence alignment of DBD segments from CaSfl1 and fungal homologs (Fig. 1A). Conserved MAPK target motifs are shaded gray, predicted phosphorylation residues are bold, and mutated residues are marked with red arrows. (**B**) Hyphal initiation of *sfl1* and the *sfl1* mutant transformed with *SFL-GFP* and *SFL1-GFP* with indicated PKA and MAPK mutations. Overnight cultures were inoculated by 1:50 dilution into YPD pH4 at 37°C and grown for 1.5 h. Images are representative of three independent experiments; percentages are means ± SD of 300 cells from triplicate experiments. *P < 0.01; NS, *P* > 0.05. (**C**) Cells from panel B are also used for gene expression analysis, as described in Fig. 1C. The data are from four biological replicates each with three technical replicates for wild type, *sfl1* or *sfl1 with SFL1* and two biological replicate for *sfl1* with *SFL1^MAPK DD^* or with *SFL1^MAPK AA^*.

Our previous study linked Sfl1 regulation to stress-responsive MAP kinases (20). Using the Prediction of Protein Kinase-specific Phosphorylation Site (PPSP) program (44), we identified potential MAPK phosphorylation sites in Sfl1. We focused on two sites within its DBD (Fig. 4A), as the DBDs of Sfl1 and Sfl2 differentiate their functions in filamentous growth (16). Furthermore, an LIWW consensus sequence, a docking domain for MAP kinases (45), precedes the predicted MAPK phosphorylation site S137 (Fig. 4A). This LIWWSP motif is conserved in Sfl1 proteins across several fungi (Fig. 4A) and is required for ScSfl1 activity in invasive growth in *S. cerevisiae* (31).

To determine whether these predicted phosphorylation sites play a role in hyphal initiation, we constructed *SFL1^S137D,T146D^* (*SFL1^MAPK DD^*) and *SFL1^S137A,T146A^* (*SFL1^MAPK AA^*) under the control of the *ADH1* promoter and transformed the constructs into an *sfl1* deletion mutant. *SFL1^MAPK DD^*-expressing cells failed to suppress hyphal initiation compared to *SFL1*-expressing cells (Fig. 4B, top row), exhibiting higher *ALS3* and *UME6* expression and lower *NRG1* expression during hyphal initiation (Fig. 4C). This is consistent with a loss of Sfl1 repressor activity. *SFL1^MAPK AA^* expressing cells showed similar cell morphology and gene expression to *SFL1*-expressing cells, indicating that the alanine substitutions at S137 and T146 did not alter Sfl1 activity (Fig. 4B and C).

To evaluate the interplay between PKA and MAPK signaling on Sfl1, we generated double mutations with alanine or aspartic acid at the predicted phosphorylation sites for both kinases. The double mutant *SFL1 ^MAPK DD, PKA A^* behaved similar to the single mutant *SFL1 ^MAPK DD^* (Fig. 4B), indicating that aspartic acid substitutions at the predicted MAPK sites can relieve Sfl1 suppression of hyphal initiation even when Sfl1 is not phosphorylatable by PKA.

All *ADH1-SFL1* constructs contained a C-terminal GFP tag and colocalized with DAPI, as previously reported (data not shown) (18). We observed distinct fluorescence patterns among the different mutant strains. Strains containing *SFL1^MAPK DD^* mutations showed reduced fluorescence in overnight cultures (Fig. 4B, framed), suggesting decreased Sfl1 protein levels that may account for the loss of Sfl1 repressor activity in these strains. In contrast, *SFL1^MAPK AA^* strains maintained fluorescence levels similar to wild type, and Sfl1 activity in these strains remained dependent on PKA site modifications (Fig. 4B, right panels). Importantly, fluorescence levels were comparable between *SFL1^PKA D^* and *SLF1^PKA A^* strains, indicating that PKA phosphorylation affects Sfl1 DNA-binding activity rather than protein stability. These results suggest that phosphorylation at either the predicted PKA or MAPK sites is sufficient to relieve Sfl1-mediated repression of hyphal initiation.

 CaSfl1 is known to be unstable (46). Our genetic data suggest that phosphorylation of CaSfl1 at S137 and/or T146 is sufficient to induce its degradation, which would predict that phosphorylation at these sites would be difficult to detect. This prediction is supported by phosphoproteomic studies of the orthologous ScSfl1 protein, where the corresponding predicted MAPK phosphorylation site T89 was not detected as phosphorylated, despite identification of phosphorylation at other ScSfl1 sites (39).

### MAPKs modulate Sfl1 stability

**Fig 5:**
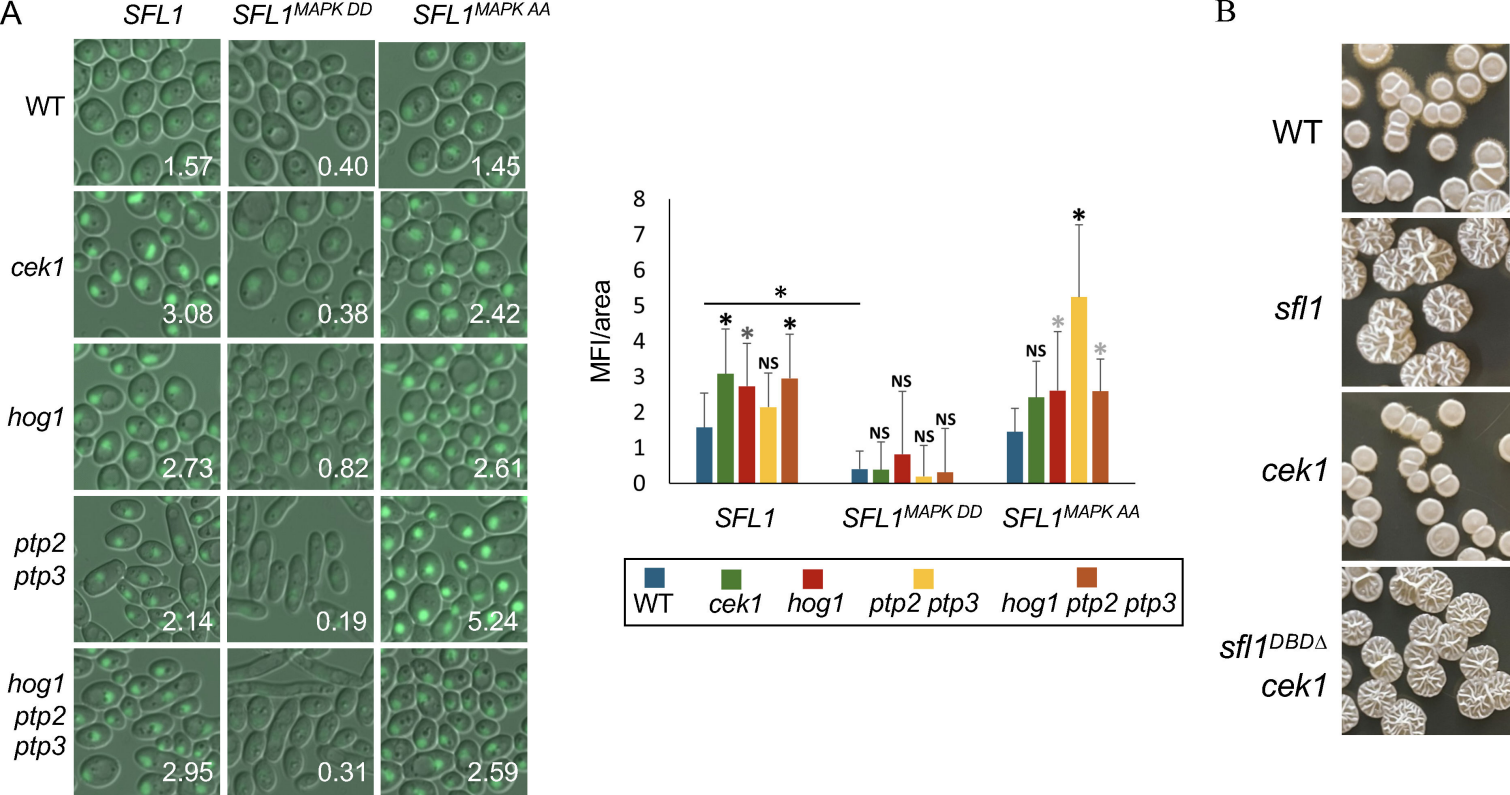
MAPKs modulate Sfl1 stability. (**A**) Fluorescence/DIC imaging of *ADH1*-driven *SFL1*-GFP constructs in indicated strains. Quantification of mean fluorescence intensity (MFI) of 30 cells with ImageJ is shown on each image (right panel). *P*-values were calculated using one-way ANOVA. *, *P* ≤ 0.01; gray *, P ≤ 0.05. (**B**) Colony morphology of indicated strains on solid Spider medium grown at 30°C for 7 days.

To examine which MAPK is responsible for Sfl1 degradation, GFP-tagged *SFL1* and *SFL1* with mutations at predicted MAPK phosphorylation sites (S137 and T146) were transformed into *cek1*, *hog1, ptp2 ptp3*, and *ptp2 ptp3 hog1* mutants. Sfl1 protein levels were estimated by quantifying GFP fluorescence from cells in overnight cultures (Fig. 5A). Strains expressing Sfl1^S137D, T146D^-GFP (Sfl1^MAPK DD^) all showed minimal signal. However, Sfl1^S137A, T146A^-GFP showed similar GFP levels to Sfl1-GFP, indicating that additional regulation occurs through other sites for Sfl1 degradation, as suggested by other studies (46, 47).

The *cek1* mutant showed slightly higher Sfl1-GFP levels than the wild-type strain, and Sfl1^S137A, T146A^-GFP levels were not significantly different between the wild-type and the *cek1* mutant (Fig. 5A). This result is consistent with the notion that Cek1 may regulate Sfl1 stability through S137 and/or T146. To examine whether Cek1 functions upstream of Sfl1, we constructed an *sfl1^DBD∆^ cek1* double mutant. Spider medium was used to assay filamentous growth as the *cek1* mutant is unable to form hyphal colonies on Spider medium (48). The *sfl1^DBD∆^ cek1* mutant displayed wrinkled colonies, similar to the *sfl1^DBD∆^* mutant, whereas the *cek1* mutant showed smooth colonies (Fig. 5B). This demonstrates that *SFL1* deletion bypasses the Cek1 requirement for wrinkled colony formation (a proxy for hyphal growth) on Spider medium.

Sfl1^S137A, T146A^-GFP in the *ptp2 ptp3* mutant showed higher GFP levels than that of the wild-type control (Fig. 5A), whereas the GFP levels were similar in the *hog1 ptp2 ptp3* triple mutant and the *hog1* mutant. This suggests that Hog1 is the major target of the tyrosine phosphatases Ptp2 and Ptp3 in modulating the stability of Sfl1^S137A, T146A^.

### Mutations at the predicted sites for PKA phosphorylation or calcineurin-binding regulate Sfl2 activity

**Fig 6:**
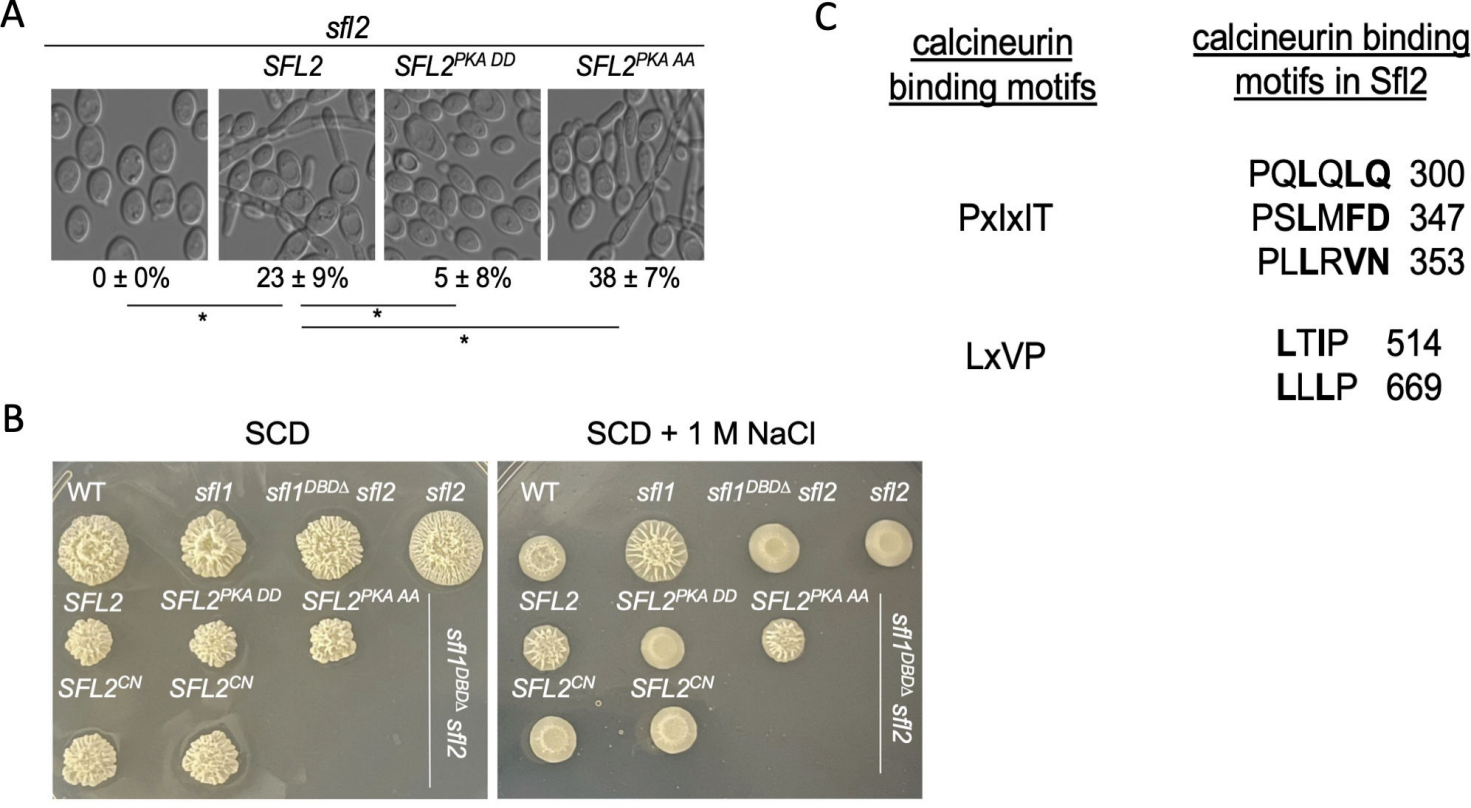
Mutations at the predicted PKA or calcineurin sites regulate Sfl2 activity. (**A**) Cell morphology of *sfl2,* or the *sfl2* mutant transformed with *SFL2* or *SFL2* with PKA mutations. Cells were grown overnight in YPD at 30°C. Images are representative of three independent experiments; percentages are means ± SD of 300 cells from triplicate experiments. *P < 0.01. (**B**) Colony morphology of WT*, sfl1*, *sfl1^DBD∆^ sfl2, and sfl2* (top row), and *sfl1^DBD∆^ sfl2* mutant transformed with *ADH1p-SFL2* or *SFL2* with PKA or calcineurin docking site (CN) mutations (bottom two rows). Cells were grown on solid SCD medium with and without 1 M NaCl at 37°C for 5 days. Images are representative of three independent experiments. (**C**) Predicted calcineurin docking SLiMs (Short Linear Motifs) based on PxIxIT and LxVP motifs (49, 50). Bolded residues were mutated to alanine.

Sfl2 is highly homologous to Sfl1 in its DBD. Like Sfl1, Sfl2 has two predicted PKA phosphorylation sites at the corresponding positions in its DBD (Fig. 1A). *SFL2* was mutated at the predicted PKA sites to *SFL2^S133D, S134D^* (*SFL2^PKA DD^*) and *SFL2^S133A, S134A^* (*SFL2^PKA AA^*), cloned under the *ADH1* promoter, and transformed into the *sfl2* mutant. Their effects on filamentation were examined after overnight growth at 30°C as overexpressing *SFL2* can promote filamentous growth at low temperature (16). The *SFL2*- or *SFL2^PKA AA^*-expressing cells generated hyphae in overnight cultures, while the *sfl2* mutant remained in yeast form (Fig. 6A). Compared to *SFL2-*expressing cells, *SFL2^PKA DD^ -*expressing cells showed a reduced percentage of hyphae while *SFL2^PKAAA^*-expressing cells exhibited an increased percentage of hyphae (Fig. 6A). Similar phenotypes were observed in *sfl1* or *sfl1^DBD∆^ sfl2* backgrounds (Fig. S2A).

We previously showed that NaCl inhibits hyphal growth, and *sfl1* can bypass this inhibition (20). Consistent with these observations, after growth on solid SCD media, NaCl inhibited filamentation (wrinkled colonies) in the wild-type strain, but not the *sfl1* mutant. The *sfl1^DBD∆^ sfl2* and *sfl2* mutants produced smooth colonies only on medium with 1 M NaCl, suggesting that filamentation under salt stress requires Sfl2 (Fig. 6B).

Unlike Sfl1, Sfl2 does not have predicted MAPK phosphorylation sites at the corresponding positions in its DBD (Fig. 4A). As calcineurin is implicated in adaptation to salt stress (51)**,** we investigated its potential role in regulating Sfl2. Calcineurin recognizes two types of short linear motifs (SLiMs) in its substrates: PxIxIT and LxVP (Fig. 6C) (49, 50, 52). Sfl2 has five predicted SLiMs (Fig. 6C). To determine whether these motifs are important for Sfl2 function, we mutated the bolded residues in the SLiMs of Sfl2 to alanine (designated *SFL2^CN^*), an approach used for functional verification of calcineurin targets (49, 50) (Fig. 6C). The *SFL2^CN^* construct was placed under the *ADH1* promoter and transformed into an *sfl1^DBD^****^∆^*** *sfl2* double mutant. *SFL2^CN^* did not complement the *sfl2* defect and was unable to promote filamentous colony formation (Fig. 6B). *SFL2^CN^*-expressing cells also showed a reduced percentage of hyphae compared to *SFL2-* or *SFL2^S133A, S134A^-*expressing cells in NaCl containing liquid medium (Fig. S2B). The controls *SFL2* and *SFL2^S133A, S134A^* complemented the *sfl2* defect, whereas *SFL2^S133D, S134D^* did not (Fig. 6B). These results suggest that Sfl2 is likely a target of calcineurin, and this regulation is important for Sfl2 function under salt stress. Calcineurin docking motifs are also found in *C. dubliniensis* Sfl2 (Fig. S2C), but not in the *S. cerevisiae* Sfl2 ortholog Mga1 (52).

## DISCUSSION

**Fig 7:**
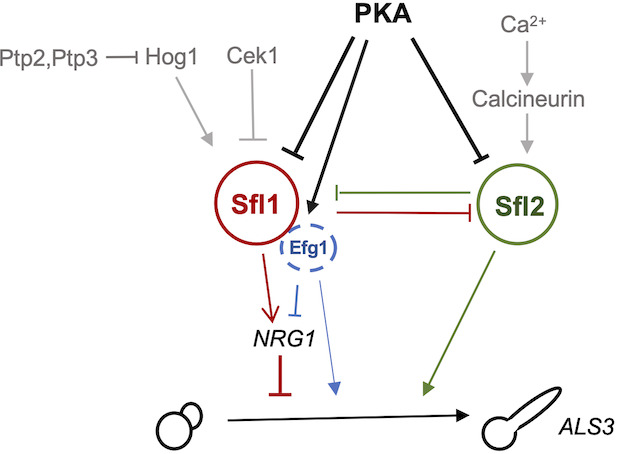
Schematic diagram for the regulation of hyphal development by PKA, MAP kinases, and calcineurin via Sfl1 and Sfl2. Arrow indicates activation while bar indicates inhibition.

Here, we provide genetic evidence that Sfl1 and Sfl2 integrate PKA, MAP kinase, and calcineurin signals to control hyphal gene expression via both PKA-dependent and PKA-independent pathways (Fig. 7).

Hyphal initiation is critical for proper hyphal development under all conditions (5). The molecular mechanisms by which PKA initiates the hyphal transcription program in *C. albicans* remain incompletely understood. In *S. cerevisiae*, the transcriptional regulators Flo8 and Sfl1 antagonistically control expression of the cell adhesin Flo11 through a shared promoter element. *In vitro* protein biochemistry experiments have demonstrated that Tpk2-mediated phosphorylation of both Flo8 and Sfl1 promotes Sfl1 dissociation from the *FLO11* promoter while simultaneously enhancing Flo8 binding (27). We suggest that PKA operates an analogous molecular switch in *C. albicans* to control hyphal initiation. Our mutational analysis supports this model: serine-to-alanine (S-to-A) substitutions at conserved PKA phosphorylation sites in Sfl1 or Sfl2 activated their function, whereas serine-to-aspartate (S-to-D) mutations (mimicking phosphorylation) inhibited their activity. Our genetic data are consistent with published *in vitro* biochemical results (27), collectively suggesting that PKA phosphorylation promotes dissociation of Sfl1 and its orthologs from target promoters. Consistent with this molecular switch model, CaFlo8 exhibits increased association with hypha-specific gene promoters during hyphal development (53), and PKA regulation of Flo8 appears conserved between *S. cerevisiae* and *C. albicans* (54–56). Additionally, Sfl1 interacts with Efg1 and other co-factors on promoter DNA to repress or activate the expression of hyphal regulators (15). This study provides evidence that the predicted PKA phosphorylation sites in Efg1 are required for hyphal initiation and *NRG1* downregulation, but not for *UME6* induction. Collectively, our genetic data support a model, wherein PKA activity differentially regulates hyphal morphogenesis through distinct mechanisms: relief of Sfl1-mediated repression triggers hyphal initiation under high PKA activity, while Sfl2 mediates hyphal elongation under low PKA activity (Fig. 7).

Environmental stress responses also regulate Sfl1 and Sfl2 through distinct post-translational mechanisms. Our results demonstrate that phosphomimetic mutations at predicted MAPK phosphorylation sites S137 and/or T146 are sufficient to reduce Sfl1 stability. Based on Sfl1-GFP levels and filamentation phenotypes observed in single and double mutants in this and published studies (20–22), we propose that the MAPK Cek1 promotes Sfl1 degradation through phosphorylation of S137 and/or T146. We further show that phosphomimetic mutation at S137 and/or T146 suppresses the phenotype of a non-phosphorylatable mutation at the predicted PKA site of Sfl1, consistent with the notion that Cek1 functions in parallel to PKA in filamentation (Fig. 7). Beyond MAPK signaling, Sfl1 is subject to additional kinase regulation. A recent study identified Sfl1 as a candidate target of the DYRK1-like kinases Yak1 and Orf19.384, with both *yak1* and *orf19.384* mutant strains exhibiting similar alterations in Sfl1 modification and increased Sfl1 protein abundance (47). Notably, Yak1 functions within the PKA pathway (57) and requires the transcription factors Efg1 and Flo8 for filamentation induction (58), suggesting potential crosstalk between these regulatory networks. Unlike Sfl1, Sfl2 does not contain equivalent predicted MAPK or Yak1 phosphorylation consensus sites (47). However, Sfl2 contains several predicted calcineurin-binding motifs that we show are critical for hyphal elongation under salt stress conditions. Calcineurin-binding motifs allow identification of new substrates, and this has led to the discovery that conserved kinase-phosphatase modules, including MAPKs and PKA paired with calcineurin, are maintained across species despite evolutionary network rewiring (52). Sfl2 appears to be a newly evolved calcineurin target.

Sfl1 and Sfl2 function as antagonistic transcription factors that reciprocally repress each other’s expression (15), forming a regulatory toggle switch that enables *C. albicans* to respond to varying PKA activity levels during hyphal morphogenesis. Under high PKA activity, relief of Sfl1-mediated repression triggers hyphal initiation, while under low PKA activity, Sfl2 promotes hyphal elongation when combined with appropriate environmental stress signals. Other PKA-independent mechanisms for hyphal development include Brg1-mediated *NRG1* repression and induction of hypha-specific genes in response to serum, N-acetylglucosamine, or reduced TOR signaling (6, 9, 59), and Sok1-mediated degradation of Nrg1 under normoxic conditions (8, 60). This integration of diverse regulatory mechanisms enables *C. albicans* to fine-tune its morphogenetic responses to complex environmental conditions in different host niches (7, 60–62).

## MATERIALS AND METHODS

### Plasmid and strain construction

Yeast strains, primers and oligos, and plasmids used in this study are listed in Tables S1 through S3, respectively.

ADH1p-*SFL2*-GFP (HLp1279) was constructed by Gibson assembly of a fragment made by PCR of *SFL2* from genomic DNA with primers 21 and 12, in frame with GFP from PCR of pYGFP3 (HLp471) with primers 21 and 22 (63) which incorporated an Sph1 site between the pieces. These were assembled into vector pBA1 (HLp549) (54, 64), cut by ClaI and KpnI (sites restored), which provided the 5′ ADH1 promoter. This plasmid was digested with AscI and transformed into the indicated *C. albicans* strain using *URA3* selection for incorporation at the *ADE2* locus.

ADH1p-*SFL1*-GFP (HLp1280) was constructed by Gibson assembly of a fragment made by PCR of genomic *SFL1* (from SN250 strain) with primers 1 and 3 and digested, gel-purified vector from HLp1279 with EcoRV (site is just upstream of *SFL2*) and SphI. This plasmid was digested with AscI and transformed into the indicated *C. albicans* strain using *URA3* selection for incorporation at the *ADE2* locus.

Plasmids with point mutations in *SFL1* or *SFL2* were made by site-directed mutagenesis using Gibson assembly with two overlapping fragments from PCR of genomic DNA (from SN250 strain) with outer primers 1 and 3 for *SFL1* or outer primers 11 and 12 for *SFL2* and inner mutation site primers as indicated for HLp1281 *SFL1^S225D^*, primers 2 and 4; HLp1282 *SFL1^S225A^,* primers 5 and 6; HLp1283 *SFL1*^S137D,T146D^, primers 7 and 8; HLp1284 *SFL1*^S137A,T146A^, primers 9 and 10, HLp1285, primers 13 and 14; HLp1286, primers 15 and 16. For double mutant plasmids, point mutations in Sfl1 were made by site-directed mutagenesis using Gibson assembly with two fragments from PCR of HLp1284/HLp1283, with outer primers 1 and 3 and mutation primers as indicated for HLp1287/1289, primers 2 and 4; HLp1288/1290, primers 5 and 6. To construct the plasmids, these PCR fragments were assembled into a gel purified vector from HLp1279, digested with EcoRV and SphI. These plasmids were digested with AscI and transformed into the indicated *C. albicans* strain using *URA3* selection for incorporation at the *ADE2* locus.

The calcineurin mutant plasmid, HLp1292, was constructed with five fragments from PCR of genomic *SFL2* with primer sets: 12 and 42, 43 and 44, 45 and 46, 47 and 48, 49 and 11. The PCR generated fragments were Gibson assembled into a gel-purified vector backbone from HLp1279 and digested with EcoRV and SphI. Calcineurin docking short linear motifs (SLiMs) were identified by manual search based on PxIxIT and LxVP motifs using the consensus [PI]x[IVLF]x[IVLF]X[TSHDEQNKR] and Lx[LIV]P (49, 50). The following residues of Sfl2 are mutated to alanine: L297, L299, Q300, L344, F346, D347, L350, V352, N353, L511, I513, L666, L668 in the assembled *SFL2^CN^* construct. This plasmid was digested with AscI and transformed into the indicated *C. albicans* strain using *URA3* selection for incorporation at the *ADE2* locus.

The *EFG1^T206A^*^,^*^T207A^*^,^*^T208A^* (HLp893) plasmid was constructed by two-step PCR amplification with two primer sets, primers 38 and 39 and primers 40 and 41 were used to amplify overlapping *EFG1* fragments with the mutation in the overlapping region as well as an SphI site. The resulting PCR products were purified and mixed as templates for another round of PCR amplifications with primers 38 and 40 which produced the full-length *EFG1^T206A^*^,^*^T207A^*^,^*^T208A^* sequence. The resulting mutant, *EFG1^T206A^*^,^*^T207A^*^,^*^T208A^*, was cloned into the BamHI-MluI site of the plasmid HLp695 replacing the wild-type copy. These were integrated at the native promoter in the *efg1* control strain HLy1881, as previously described (65).

All constructs generated above are confirmed by DNA sequencing of inserts from plasmid DNA.

CRISPR deletion of the *SFL1* DNA-binding domain (*sfl1^DBD∆^*) utilized dDNA oligonucleotide 163. This was transformed along with MSSI digested “intact” gRNA plasmid (HLp1291: annealed oligos 36 and 37 Gibson cloned into “entry vector” (PCR of pADH100 (HLp1263) with AHO1098 and AHO1099) and plasmid pADH99 (HLp1262) (41).

All *C. albicans* strains were adjusted as appropriate for auxotrophy by addition of *URA3* at the *ADE2* locus from HLp549/pBES116 digested with AscI or addition of *ARG4* at the native locus by PCR of genomic DNA with primers 33 and 34 via transformation. Cells were streaked to FOA medium (0.1 g/100 mL) for removal of *URA3*.

Sequence alignments were made by Clustal Omega (1.2.4) (66–68).

### Media, growth conditions

Overnight *C. albicans* saturated cultures were grown in YPD at 30°C and were diluted for hypha-induction 1:50 into prewarmed YPD media at 37°C in a water bath with 180 rpm and supplemented with HCl for pH 4 as indicated. Colony morphology assay on SCD with and without 1 M NaCl was performed by plating 1.2 × 10^6^ cells per colony (69). Single cell-colony morphology assay was performed by plating to solid spider medium at a density of 50–100 cells per plate (48). Statistical analysis was performed by unpaired *t* test using Graphpad Quickcalcs Website: https://www.graphpad.com/quickcalcs/ttest1/.

### Cell imaging and quantification of fluorescence signal

Cells from aliquots of cultures were visualized in their test media on an inverted Zeiss Axio Observer.Z1 microscope (Carl Zeiss MicroImaging, Inc., Thornwood, NY) by DIC or with a fluorescent system equipped with X-Cite series 120 mercury lamps using a GFP filter (9 sec. exp.). Images were taken using a ×100 numerical aperture 1.4 objective lens.

Processing was done using the software ImageJ (National Institutes of Health, USA). Mean fluorescence intensity (MFI) per unit area was determined based on randomly selected images with at least 30 cells in total per strain by ImageJ. Values were calculated as the intensity density of the GFP in a cell, minus the background signal, over the total cell area based on DIC images. *P*-values were calculated using one-way ANOVA Calculator including Tukey HSD *post hoc* analysis Social Science Statistics website: https://www.socscistatistics.com/tests/anova/default2.aspx.

### Real-time qRT-PCR

Overnight *C. albicans* saturated cultures were grown in YPD at 30°C (OD_600_ = 16–18) and were diluted for hypha induction 1:50 into prewarmed YPD media at 37°C in a water bath with 180 rpm and supplemented with HCl for pH 4 as indicated. Cells were harvested at 4°C, and RNA was extracted using the Qiagen RNeasy Kit and 0.2 µg of total RNA was reverse transcribed into cDNA using the BioRad iScript cDNA synthesis kit. Experiments were performed in biological duplicate, and trends were consistent across experiments. Quantitative PCR was performed on a BioRad iCycler using BioRad iTaq Universal SYBR Green Supermix with the following: *CDC28* primers 23 and 24, *UME6* primers 27 and 28, *ALS3* primers 29 and 30, *NRG1* primers 25 and 26. The cycle parameters were 95°C for 1 min, then 39 cycles of 95 °C for 10 s, 56°C for 45 s, and 68°C for 20 s. The data show the average of two or more independent quantitative RT-PCR experiments, with error bars representing the SEM (standard deviation divided by square root of sample size; measures how much discrepancy is likely in a sample’s mean compared with the population mean).

### Structural modeling

Predicted protein structure was obtained from AlphaFold website: https://alphafold.ebi.ac.uk/entry/Q5A287 (version 2.3.2) (70) and visualized using Firstglance in Jmol website: Firstglance.jmol.org (version 4.1).

## SUPPLEMENTAL MATERIAL

Tables S1 to S3 msphere.00689-25-s0001.docx *C. albicans* strains, plasmids, and primers used in this study.

Fig. S1 and S2 msphere.00689-25-s0002.docx Sfl1 DBD structure and morphology of SFL2 PKA and calcineurin-binding mutants.

ASM does not own the copyrights to Supplemental Material that may be linked to, or accessed through, an article. The authors have granted ASM a non-exclusive, world-wide license to publish the Supplemental Material files. Please contact the corresponding author directly for reuse.
