# Supplementary material for: Regulation of hyphal development by protein kinase A, stress-responsive MAP kinases, and calcineurin via transcription factors Sfl1 and Sfl2 in Candida albicans
Source: mSphere. 2026 Jan 8;11(2):e00689-25. doi: 10.1128/msphere.00689-25 (PMC12931275; doi:10.1128/msphere.00689-25)
Supplement: Tables S1 to S3 — C. albicans strains, plasmids, and primers used in this study. [file msphere.00689-25-s0001.docx]

| HLy#  Table S1. *C. albicans* strains used in this study | Strain | Parent strain | Genotype | Source |
| --- | --- | --- | --- | --- |
|  | SN250 |  | *ura3Δ-iro1Δ::imm^434^/URA3-IRO1, his1Δ/his1Δ, arg4Δ/arg4Δ, leu2Δ::C.m.LEU2/leu2Δ::C.d.HIS1* | [6] |
| HLy4599 | SN250 + *ARG4* |  | *ARG4*/*arg4* *leu2::HIS1*/*leu2* *:: LEU2 his1*/*his1*  *ura3::1 imm434/ura3::1 imm434* | This study |
|  | CAI4 |  | *ura3::1 imm^434^/ura3::1 imm^434^* | [1] |
|  | *sfl1* | SN152 | *ura3Δ-iro1Δ::imm^434^/URA3-IRO1, his1Δ/his1Δ, arg4Δ/arg4Δ., leu2Δ/leu2Δ, sfl1Δ::HIS1/sfl1Δ::LEU2* | [3] |
| HLy4600 | *sfl1* *ARG4* | *sfl1* | *ura3Δ-iro1Δ::imm^434^/URA3-IRO1, his1Δ/his1Δ, arg4Δ/ARG4, leu2Δ/leu2Δ, sfl1Δ::HIS1/sfl1Δ::LEU2* | This study |
| HLy4661 | *sfl1 ARG4 -ura3* | HLy4600 | *ura3Δ-iro1Δ::imm^434^/ ura3Δ-iro1Δ::imm^434^, his1Δ/his1Δ, arg4Δ/ARG4,leu2Δ/leu2Δ, sfl1Δ::HIS1/sfl1Δ::LEU2* |  |
|  | *sfl2* | SN152 | *ura3Δ-iro1Δ::imm^434^/URA3-IRO1, his1Δ/his1Δ, arg4Δ/arg4Δ, leu2Δ/leu2Δ, sfl2Δ::HIS1/sfl2Δ::LEU2* | [3] |
| HLy4602 | *sfl2 ARG4* | *sfl2* | *ura3Δ-iro1Δ::imm^434^/URA3-IRO1, his1Δ/his1Δ, arg4Δ/ARG4, leu2Δ/leu2Δ, sfl2Δ::HIS1/sfl2Δ::LEU2* | This study |
| HLy4660 | *sfl2 ARG4 -ura3* | HLy4602 | *ura3Δ-iro1Δ::imm^434^/ura3Δ-iro1Δ::imm^434^, his1Δ/his1Δ, arg4Δ/ARG4, leu2Δ/leu2Δ, sfl2Δ::HIS1/sfl2Δ::LEU2* | This study |
| HLy3593 | *tpk2* | CAI4 | *ura3::imm434/ura3::imm434 tpk2Δ::hisG/tpk2Δ::hisG-URA3-hisG* | [7] |
|  | *efg1* | SN152 | *ura3Δ-iro1Δ::imm^434^/URA3-IRO1, his1Δ/his1Δ, arg4Δ/arg4Δ, leu2Δ/leu2Δ, efg1Δ::HIS1/efg1Δ::LEU2* | [3] |
| HLy4601 | *efg1* + *ARG4* | *efg1* | *ura3Δ-iro1Δ::imm^434^/URA3-IRO1, his1Δ/his1Δ, arg4Δ/ARG4, leu2Δ/leu2Δ, efg1Δ::HIS1/efg1Δ::LEU2* | This study |
|  | *cek1* | SN152 | *ura3Δ-iro1Δ::imm^434^/URA3-IRO1, his1Δ/his1Δ, arg4Δ/arg4Δ, leu2Δ/leu2Δ, cek1Δ::HIS1/cek1Δ::LEU2* | [6] |
| HLy4632 | *cek1*  *ARG4* | *cek1* | *ura3Δ-iro1Δ::imm^434^/URA3-IRO1, his1Δ/his1Δ, arg4Δ/ARG4, leu2Δ/leu2Δ, cek1Δ::HIS1/cek1Δ::LEU2* | This study |
| HLy4659 | *cek1 -ura3* | HLy4632 | *ura3Δ-iro1Δ::imm^434^/ura3Δ-iro1Δ::imm^434^, his1Δ/his1Δ, arg4Δ/ARG4, leu2Δ/leu2Δ, cek1Δ::HIS1/cek1Δ::LEU2* | This study |
|  | *hog1* | SN152 | *ura3Δ-iro1Δ::imm^434^/URA3-IRO1, his1Δ/his1Δ, arg4Δ/arg4Δ, leu2Δ/leu2Δ, hog1Δ::HIS1/hog1Δ::LEU2* | [6] |
| HLy4662 | *hog1 ARG4* | *hog1* | *ura3Δ-iro1Δ::imm^434^/URA3-IRO1, his1Δ/his1Δ, arg4Δ/ARG4, leu2Δ/leu2Δ, hog1Δ::HIS1/hog1Δ::LEU2* | This study |
| HLy4658 | *hog1 ARG4 -ura3* | HLy4662 | *ura3Δ-iro1Δ::imm^434^/ura3Δ-iro1Δ::imm^434^, his1Δ/his1Δ, arg4Δ/ARG4, leu2Δ/leu2Δ, hog1Δ::HIS1/hog1Δ::LEU2* | This study |
| HLy4101 | *ptp2ptp3* | SN152 | *ura3Δ-iro1Δ::imm^434^/URA3-IRO1, his1Δ/his1Δ, arg4Δ/arg4Δ, leu2Δ/leu2Δ, ptp3Δ::C.dubliniensis HIS/ptp3Δ::C.maltosa LEU2, ptp2Δ::ARG4/ptp2::ARG4* | [9] |
| HLy4105 | *hog1ptp2ptp3* | SN152 | *ura3Δ-iro1Δ::imm^434^/ ura3Δ-iro1Δ::imm^434^, his1Δ/his1Δ, arg4Δ/arg4Δ, leu2Δ/leu2Δ, ptp3Δ::C.dubliniensis HIS/ptp3Δ::C.maltosa LEU2, ptp2 Δ::ARG4/ptp2::ARG4, hog1Δ::URA3/hog1Δ::FRT* | [9] |
| HLy1881 | *efg1* | CAI4 | *ura3Δ::1 imm434/ura3Δ::1 imm434 efg1::hisG/efg1::higG-URA3-hisG* | [8] |
| HLy3499 | *efg1/EFG1* | HLy1881 | *ura3::1 imm434/ura3::1 imm434*  *efg1::hisG/EFG1-MYC -URA3* | [10] |
| HLy3489 | *efg1/EFG1^T206A,T207A, T208A^* | HLy1881 | *ura3::1 imm434/ura3::1 imm434*  *efg1::hisG/EFG1 ^T206A,T207A, T208A^-MYC -URA3* | This study |
| HLy4595 | *sfl1 ^DBD∆^tpk2* | *tpk2* | *ura3::imm434/ura3::imm434 tpk2Δ::hisG/tpk2Δ::hisG-URA3-hisG, sfl1^DBD∆^/sfl1^DBD∆^* | This study |
| HLy4596 | *sfl ^DBD∆^*  *EFG1^T206A, T207A, T208A^* | *EFG1^T206A, T208A^* | *ura3::1 imm434/ura3::1 imm434 efg1::hisG/EFG1 ^T206A,T207A, T208A^-MYC -URA3, sfl1^DBD∆^/sfl1^DBD∆^* | This study |
| HLy4597 | *sfl ^DBD∆^cek* | HLy4632 | *ura3Δ-iro1Δ::imm^434^/URA3-IRO1, his1Δ/his1Δ, arg4Δ/ARG4, leu2Δ/leu2Δ, cek1Δ::HIS1/cek1Δ::LEU2, sfl1^DBD∆^/sfl1^DBD∆^* | This study |
| 4604 | *sfl2 ARG4*  ADH1pSFL2GFP | HLy4660 | *ura3Δ-iro1Δ::imm^434^/ura3Δ-iro1Δ::imm^434^, his1Δ/his1Δ, arg4Δ/ARG4, leu2Δ/leu2Δ, sfl2Δ::HIS1/sfl2Δ::LEU2, ADE2/ade2::ADH1pSFL2GFP*-*URA3* | This study |
| 4605 | *sfl1 ARG4* ADH1pSFL2GFP | HLy4661 | *ura3Δ-iro1Δ::imm^434^/ ura3Δ-iro1Δ::imm^434^, his1Δ/his1Δ, arg4Δ/ARG4,leu2Δ/leu2Δ, sfl1Δ::HIS1/sfl1Δ::LEU2, ADE2/ade2::ADH1pSFL2GFP-URA3* | This study |
| 4606 | *sfl1 ARG4 ADH1pSFL1GFP* | HLy4661 | *ura3Δ-iro1Δ::imm^434^/ ura3Δ-iro1Δ::imm^434^, his1Δ/his1Δ, arg4Δ/ARG4,leu2Δ/leu2Δ, sfl1Δ::HIS1/sfl1Δ::LEU2, ADE2/ade2::ADH1pSFL1GFP-URA3* | This study |
| 4607 | *sfl1 ARG4 ADH1pSFL1^S225D^GFP* | HLy4661 | *ura3Δ-iro1Δ::imm^434^/ ura3Δ-iro1Δ::imm^434^, his1Δ/his1Δ, arg4Δ/ARG4,leu2Δ/leu2Δ, sfl1Δ::HIS1/sfl1Δ::LEU2, ADE2/ade2::ADH1pSFL1^S225D^GFP-URA3* | This study |
| 4608 | *sfl1 ARG4 ADH1pSFL1^S225A^GFP* | HLy4661 | *ura3Δ-iro1Δ::imm^434^/ ura3Δ-iro1Δ::imm^434^, his1Δ/his1Δ, arg4Δ/ARG4,leu2Δ/leu2Δ, sfl1Δ::HIS1/sfl1Δ::LEU2, ADE2/ade2::ADH1pSFL1^S225A^GFP-URA3* | This study |
| 4609 | *sfl1 ARG4 ADH1pSFL1^S137D, T146D^GFP* | HLy4661 | *ura3Δ-iro1Δ::imm^434^/ ura3Δ-iro1Δ::imm^434^, his1Δ/his1Δ, arg4Δ/ARG4,leu2Δ/leu2Δ, sfl1Δ::HIS1/sfl1Δ::LEU2, ADE2/ade2:: ADH1pSFL1^S137D, T146D^GFP-URA3* | This study |
| 4610 | *sfl1 ARG4*  *ADH1pSFL1^S137A, T146A^GFP* | HLy4661 | *ura3Δ-iro1Δ::imm^434^/ ura3Δ-iro1Δ::imm^434^, his1Δ/his1Δ, arg4Δ/ARG4,leu2Δ/leu2Δ, sfl1Δ::HIS1/sfl1Δ::LEU2, ADE2/ade2::ADH1pSFL1^S137A, T146A^GFP-URA3* | This study |
| 4611 | *sfl1 ARG4*  *ADH1pSFL2^S133D, S134D^GFP* | HLy4661 | *ura3Δ-iro1Δ::imm^434^/ ura3Δ-iro1Δ::imm^434^, his1Δ/his1Δ, arg4Δ/ARG4,leu2Δ/leu2Δ, sfl1Δ::HIS1/sfl1Δ::LEU2, ADE2/ade2::ADH1pSFL2^S133D, S134D^GFP-URA3* | This study |
| 4612 | *sfl1 ARG4*  *ADH1pSFL2^S133A, S134A^GFP* | HLy4661 | *ura3Δ-iro1Δ::imm^434^/ ura3Δ-iro1Δ::imm^434^, his1Δ/his1Δ, arg4Δ/ARG4,leu2Δ/leu2Δ, sfl1Δ::HIS1/sfl1Δ::LEU2, ADE2/ade2::ADH1pSFL2^S133A, S134A^GFP-URA3* | This study |
| 4613 | *sfl1* *ARG4*  *ADH1pSFL1^S137A^*^,^ *^T146A^*^,^ *^S225D^GFP* | HLy4661 | *ura3Δ-iro1Δ::imm^434^/ ura3Δ-iro1Δ::imm^434^, his1Δ/his1Δ, arg4Δ/ARG4,leu2Δ/leu2Δ, sfl1Δ::HIS1/sfl1Δ::LEU2, ADE2/ade2:: ADH1pSFL1^S137A, T146A, S225D^GFP-URA3* | This study |
| 4614 | *sfl1* *ARG4*  *ADH1pSFL1^S137A^*^,^ *^T146A^*^,^ *^S225A^GFP* | HLy4661 | *ura3Δ-iro1Δ::imm^434^/ ura3Δ-iro1Δ::imm^434^, his1Δ/his1Δ, arg4Δ/ARG4,leu2Δ/leu2Δ, sfl1Δ::HIS1/sfl1Δ::LEU2, ADE2/ade2::ADH1pSFL1^S137A, T146A, S225A^GFP-URA3* | This study |
| 4615 | *sfl1* *ARG4* *ADHp1SFL1^S137D^*^, T146D,^ *^S225D^GFP* | HLy4661 | *ura3Δ-iro1Δ::imm^434^/ ura3Δ-iro1Δ::imm^434^, his1Δ/his1Δ, arg4Δ/ARG4,leu2Δ/leu2Δ, sfl1Δ::HIS1/sfl1Δ::LEU2, ADE2/ade2::ADHp1SFL1^S137D, T146D, S225D^GFP-URA3* | This study |
| 4616 | *sfl2* *ARG4*  *ADH1pSFL2^S133D^*^,^ *^S134D^GFP* | HLy4660 | *ura3Δ-iro1Δ::imm^434^/ura3Δ-iro1Δ::imm^434^, his1Δ/his1Δ, arg4Δ/ARG4, leu2Δ/leu2Δ, sfl2Δ::HIS1/sfl2Δ::LEU2, ADE2/ade2::ADH1pSFL2^S133D^*^,^ *^S134D^GFP-URA3* | This study |
| 4617 | *sfl2* *ARG4*  *ADH1pSFL2^S133A^*^,^ *^S134A^GFP* | HLy4660 | *ura3Δ-iro1Δ::imm^434^/ura3Δ-iro1Δ::imm^434^, his1Δ/his1Δ, arg4Δ/ARG4, leu2Δ/leu2Δ, sfl2Δ::HIS1/sfl2Δ::LEU2, ADE2/ade2::ADH1pSFL2^S133A^*^,^ *^S134A^GFP-URA3* | This study |
| 4618 | *sfl1 ARG4 ADH1pSFL1^S137A,T146D,S225D^GFP* | HLy4661 | *ura3Δ-iro1Δ::imm^434^/ ura3Δ-iro1Δ::imm^434^, his1Δ/his1Δ, arg4Δ/ARG4,leu2Δ/leu2Δ, sfl1Δ::HIS1/sfl1Δ::LEU2, ADE2/ade2::ADH1pSFL1^S137A,T146D,S225D^GFP-URA3* | This study |
| 4620 | *sfl1^DBD∆^sfl2* *ARG4* | HLy4602 | *ura3Δ-iro1Δ::imm^434^/URA3-IRO1, his1Δ/his1Δ, arg4Δ/ARG4, leu2Δ/leu2Δ, sfl2Δ::HIS1/sfl2Δ::LEU2 sfl1^DBD∆^/sfl1^DBD∆^* | This study |
| 4663 | *sfl ^DBD∆^ sfl2 ARG4 -ura3* | HLy4620 | *ura3Δ-iro1Δ::imm^434^/ ura3Δ-iro1Δ::imm^43^, his1Δ/his1Δ, arg4Δ/ARG4, leu2Δ/leu2Δ, sfl2Δ::HIS1/sfl2Δ::LEU2 sfl1^DBD∆^/sfl1^DBD∆^* |  |
| 4621 | *sfl1^DBD∆^sfl2* *ARG4*  *ADH1pSFL2GFP* | HLy4663 | *ura3Δ-iro1Δ::imm^434^/ ura3Δ-iro1Δ::imm^434^, his1Δ/his1Δ, arg4Δ/ARG4, leu2Δ/leu2Δ, sfl2Δ::HIS1/sfl2Δ::LEU2, sfl1^DBD∆^/sfl1^DBD∆^  ADE2/ade2::ADH1pSFL2GFP-URA3* | This study |
| 4622 | *sfl1^DBD∆^sfl2* *ARG4*  *ADH1pSFL2^S133D^*^,^ *^S134D^GFP* | HLy4663 | *ura3Δ-iro1Δ::imm^434^/ ura3Δ-iro1Δ::imm^434^, his1Δ/his1Δ, arg4Δ/ARG4, leu2Δ/leu2Δ, sfl2Δ::HIS1/sfl2Δ::LEU2, sfl1^DBD∆^/sfl1^DBD^ , ADE2/ade2::ADH1pSFL2^S133D, S134D^GFP-URA3* | This study |
| 4623 | *sfl1^DBD∆^sfl2* *ARG4*  *ADH1pSFL2^S133A^*^,^ *^S134A^GFP* | HLy4663 | *ura3Δ-iro1Δ::imm^434^/ ura3Δ-iro1Δ::imm^43^, his1Δ/his1Δ, arg4Δ/ARG4, leu2Δ/leu2Δ, sfl2Δ::HIS1/sfl2Δ::LEU2, sfl1^DBD∆^/sfl1^DBD∆^  ADE2/ade2::ADH1pSFL2^S133A, S134A^GFP-URA3* | This study |
| 4624 | *efg1*/*EFG1* *sfl1^DBD∆^* | HLy3499 | *ura3::1 imm434/ura3::1 imm434*  *efg1::hisG/EFG1-MYC -URA3 sfl1^DBD∆^/sfl1^DBD∆^* | This study |
| 4625 | *sfl1sfl2 ARG4*  *ADH1pSFL2 ^CN^* | HLy4663 | *ura3Δ-iro1Δ::imm^434^/ ura3Δ-iro1Δ::imm^43^, his1Δ/his1Δ, arg4Δ/ARG4, leu2Δ/leu2Δ, sfl2Δ::HIS1/sfl2Δ::LEU2, sfl1^DBD∆^/sfl1^DBD∆^  ADE2/ade2::ADH1pSFL2 ^CN^-URA3* | This study |
| 4626 | *sfl1sfl2 ARG4 ADH1pSFL2^CN^* | HLy4663 | *ura3Δ-iro1Δ::imm^434^/ ura3Δ-iro1Δ::imm^43^, his1Δ/his1Δ, arg4Δ/ARG4, leu2Δ/leu2Δ, sfl2Δ::HIS1/sfl2Δ::LEU2, sfl1^DBD∆^/sfl1^DBD∆^  ADE2/ade2::ADH1pSFL2 ^CN^-URA3* | This study |
| 4633 | CAI4 *ADH1pSFL1GFP* | CAI4 | *ura3::1 imm434/ura3::1 imm434, ADE2/ade2::ADH1pSFL1GFP-URA3* | This study |
| 4634 | CAI4 *ADH1pSFL1^S137D, T146D^GFP* | CAI4 | *ura3::1 imm434/ura3::1 imm434, ADE2/ade2::ADH1pSFL1^S137D, T146D^GFP-URA3* | This study |
| 4635 | CAI4 *ADH1pSFL1^S137A^*^,^ *^T146A^GFP* | CAI4 | *ura3::1 imm^434^/ura3::1 imm^434^, ADE2/ade2::ADH1pSFL1^S137A^*^,^ *^T146A^GFP-URA3* | This study |
| 4636 | CAI4 *ADH1pSFL1^S225D^GFP* | CAI4 | *ura3::1 imm^434^/ura3::1 imm^434^ ADE2/ade2::ADH1pSFL1^S225D^GFP-URA3* | This study |
| 4637 | CAI4 *ADH1pSFL1^S225A^GFP* | CAI4 | *ura3::1 imm434/ura3::1 imm434 ADE2/ade2::ADH1pSFL1^S225A^GFP-URA3* | This study |
| 4638 | *cek1 ARG4*  *ADH1pSFL1GFP* | HLy4659 | *ura3Δ-iro1Δ::imm^434^/ura3Δ-iro1Δ::imm^434^, his1Δ/his1Δ, arg4Δ/ARG4, leu2Δ/leu2Δ, cek1Δ::HIS1/cek1Δ::LEU2 ADE2/ade2::ADH1pSFL1GFP-URA3* | This study |
| 4639 | *cek1 ARG4*  *ADH1pSFL1^S137A^*^,^ *^T146A^GFP* | HLy4659 | *ura3Δ-iro1Δ::imm^434^/ura3Δ-iro1Δ::imm^434^, his1Δ/his1Δ, arg4Δ/ARG4, leu2Δ/leu2Δ, cek1Δ::HIS1/cek1Δ::LEU2, ADE2/ade2::ADH1pSFL1^S137A^*^,^ *^T146A^GFP-URA3* | This study |
| 4640 | *cek1* *ARG4*  *ADH1pSFL1^S137D^*^,^ *^T146D^GFP* | HLy4659 | *ura3Δ-iro1Δ::imm^434^/ura3Δ-iro1Δ::imm^434^, his1Δ/his1Δ, arg4Δ/ARG4, leu2Δ/leu2Δ, cek1Δ::HIS1/cek1Δ::LEU2, ADE2/ade2::ADH1pSFL1^S137D^*^,^ *^T146D^GFP-URA3* | This study |
| 4641 | *cek1 ARG4*  *ADH1pSFL1^S225D^GFP* | HLy4659 | *ura3Δ-iro1Δ::imm^434^/ura3Δ-iro1Δ::imm^434^, his1Δ/his1Δ, arg4Δ/ARG4, leu2Δ/leu2Δ, cek1Δ::HIS1/cek1Δ::LEU2, ADE2/ade2::ADH1pSFL1^S225D^GFP-URA3* | This study |
| 4642 | *cek1*  *ARG4*  *ADH1pSFL1^S225A^GFP* | HLy4659 | *ura3Δ-iro1Δ::imm^434^/ura3Δ-iro1Δ::imm^434^, his1Δ/his1Δ, arg4Δ/ARG4, leu2Δ/leu2Δ, cek1Δ::HIS1/cek1Δ::LEU2, ADE2/ade2::ADH1pSFL1^S225A^GFP-URA3* | This study |
| 4643 | *hog1 ARG4*  *ADH1pSFL1GFP* | HLy4658 | *ura3Δ-iro1Δ::imm^434^/ura3Δ-iro1Δ::imm^434^, his1Δ/his1Δ, arg4Δ/ARG4, leu2Δ/leu2Δ, hog1Δ::HIS1/hog1Δ::LEU2, ADE2/ade2::ADH1pSFL1GFP-URA3* | This study |
| 4644 | *hog1* *ARG4*  *ADH1pSFL1^S137D^*^,^ *^T146D^GFP* | HLy4658 | *ura3Δ-iro1Δ::imm^434^/ura3Δ-iro1Δ::imm^434^, his1Δ/his1Δ, arg4Δ/ARG4, leu2Δ/leu2Δ, hog1Δ::HIS1/hog1Δ::LEU2, ADE2/ade2::ADH1pSFL1^S137D^*^,^ *^T146D^GFP-URA3* | This study |
| 4645 | *hog1* *ARG4*  *ADH1pSFL1^S137A^*^,^ *^T146A^GFP* | HLy4658 | *ura3Δ-iro1Δ::imm^434^/ura3Δ-iro1Δ::imm^434^, his1Δ/his1Δ, arg4Δ/ARG4, leu2Δ/leu2Δ, hog1Δ::HIS1/hog1Δ::LEU2, ADE2/ade2::ADH1pSFL1^S137A^*^,^ *^T146A^GFP-URA3* | This study |
| 4646 | *hog1* *ARG4* *ADH1pSFL1^S225D^GFP* | HLy4658 | *ura3Δ-iro1Δ::imm^434^/ura3Δ-iro1Δ::imm^434^, his1Δ/his1Δ, arg4Δ/ARG4, leu2Δ/leu2Δ, hog1Δ::HIS1/hog1Δ::LEU2, ADE2/ade2::ADH1pSFL1^S225D^GFP-URA3* | This study |
| 4647 | *hog1* *ARG4* *ADH1pSFL1^S225A^GFP* | HLy4658 | *ura3Δ-iro1Δ::imm^434^/ura3Δ-iro1Δ::imm^434^, his1Δ/his1Δ, arg4Δ/ARG4, leu2Δ/leu2Δ, hog1Δ::HIS1/hog1Δ::LEU2, ADE2/ade2::ADH1pSFL1^S225A^GFP-URA3* | This study |
| 4648 | *ptp2ptp3* *ADH1pSFL1GFP* | HLy4101 | *ura3Δ-iro1Δ::imm^434^/URA3-IRO1, his1Δ/his1Δ, arg4Δ/arg4Δ, leu2Δ/leu2Δ, ptp3Δ::C.dubliniensis HIS/ptp3Δ::C.maltosa LEU2, ptp2Δ::ARG4/ptp2::ARG4, ADE2/ade2::ADH1pSFL1GFP-URA3* | This study |
| 4649 | *ptp2ptp3*  *ADH1pSFL1^S137D^*^,^ *^T146D^GFP* | HLy4101 | *ura3Δ-iro1Δ::imm^434^/URA3-IRO1, his1Δ/his1Δ, arg4Δ/arg4Δ, leu2Δ/leu2Δ, ptp3Δ::C.dubliniensis HIS/ptp3Δ::C.maltosa LEU2, ptp2Δ::ARG4/ptp2::ARG4, ADE2/ade2::ADH1pSFL1^S137D^*^,^ *^T146D^GFP-URA3* | This study |
| 4650 | *ptp2ptp3*  *ADH1pSFL1^S137A^*^,^ *^T146A^GFP* | HLy4101 | *ura3Δ-iro1Δ::imm^434^/URA3-IRO1, his1Δ/his1Δ, arg4Δ/arg4Δ, leu2Δ/leu2Δ, ptp3Δ::C.dubliniensis HIS/ptp3Δ::C.maltosa LEU2, ptp2Δ::ARG4/ptp2::ARG4, ADE2/ade2::ADH1pSFL1^S137A^*^,^ *^T146A^GFP-URA3* | This study |
| 4651 | *ptp2ptp3* *ADH1pSFL1^S225D^GFP* | HLy4101 | *ura3Δ-iro1Δ::imm^434^/URA3-IRO1, his1Δ/his1Δ, arg4Δ/arg4Δ, leu2Δ/leu2Δ, ptp3Δ::C.dubliniensis HIS/ptp3Δ::C.maltosa LEU2, ptp2Δ::ARG4/ptp2::ARG4, ADE2/ade2::ADH1pSFL1^S225D^GFP-URA3* | This study |
| 4652 | *ptp2ptp3* *ADH1pSFL1^S225A^GFP* | HLy4101 | *ura3Δ-iro1Δ::imm^434^/URA3-IRO1, his1Δ/his1Δ, arg4Δ/arg4Δ, leu2Δ/leu2Δ, ptp3Δ::C.dubliniensis HIS/ptp3Δ::C.maltosa LEU2, ptp2Δ::ARG4/ptp2::ARG4, ADE2/ade2::ADH1pSFL1^S225A^GFP-URA3* | This study |
| 4653 | *ptp2ptp3hog1 ADH1pSFL1GFP* | HLy4105 | *ura3Δ-iro1Δ::imm^434^/ ura3Δ-iro1Δ::imm^434^, his1Δ/his1Δ, arg4Δ/arg4Δ, leu2Δ/leu2Δ, ptp3Δ::C.dubliniensis HIS/ptp3Δ::C.maltosa LEU2, ptp2 Δ::ARG4/ptp2::ARG4, hog1Δ::URA3/hog1Δ::FRT, ADE2/ade2::ADH1pSFL1GFP-URA3* | This study |
| 4654 | *ptp2ptp3hog1 ADH1pSFL1^S137D,T146D^GFP* | HLy4105 | *ura3Δ-iro1Δ::imm^434^/ ura3Δ-iro1Δ::imm^434^, his1Δ/his1Δ, arg4Δ/arg4Δ, leu2Δ/leu2Δ, ptp3Δ::C.dubliniensis HIS/ptp3Δ::C.maltosa LEU2, ptp2 Δ::ARG4/ptp2::ARG4, hog1Δ::URA3/hog1Δ::FRT, ADE2/ade2::ADH1pSFL1^S137D,T146D^GFP-URA3* | This study |
| 4655 | *ptp2ptp3hog1*  *ADH1pSFL1^S137A,T146A^GFP* | HLy4105 | *ura3Δ-iro1Δ::imm^434^/ ura3Δ-iro1Δ::imm^434^, his1Δ/his1Δ, arg4Δ/arg4Δ, leu2Δ/leu2Δ, ptp3Δ::C.dubliniensis HIS/ptp3Δ::C.maltosa LEU2, ptp2 Δ::ARG4/ptp2::ARG4, hog1Δ::URA3/hog1Δ::FRT, ADE2/ade2::ADH1pSFL1^S137A,T146A^GFP-URA3* | This study |
| 4656 | *ptp2ptp3hog1*  *ADH1pSFL1^S225D^GFP* | HLy4105 | *ura3Δ-iro1Δ::imm^434^/ ura3Δ-iro1Δ::imm^434^, his1Δ/his1Δ, arg4Δ/arg4Δ, leu2Δ/leu2Δ, ptp3Δ::C.dubliniensis HIS/ptp3Δ::C.maltosa LEU2, ptp2 Δ::ARG4/ptp2::ARG4, hog1Δ::URA3/hog1Δ::FRT, ADE2/ade2::ADH1pSFL1^S225D^GFP-URA3* | This study |

1 Fonzi, W. A. and Irwin, M. Y. (1993) Isogenic strain construction and gene mapping in Candida albicans. Genetics 134, 717-728

2 Blankenship JR, Fanning S, Hamaker JJ, Mitchell AP. 2010. An extensive circuitry for cell wall regulation in Candida albicans. PLoS Pathog 6:e1000752.

3 Homann OR, Dea J, Noble SM, Johnson AD. 2009. A phenotypic profile of the Candida albicans regulatory network. PLoS Genet 5:e1000783.

4 Su C, Lu Y, Liu H. 2013. Reduced TOR signaling sustains hyphal development in Candida albicans by lowering Hog1 basal activity. Mol Biol Cell 24:385–97.

5 Lu, Y., Su, C., Wang, A. and Liu, H. Hyphal development in Candida albicans requires two temporally linked changes in promoter chromatin for initiation and maintenance. PLoS Biol 9, e1001105

6 Noble, S. M. and Johnson, A. D. (2005) Strains and strategies for large-scale gene deletion studies of the diploid human fungal pathogen Candida albicans. Eukaryot Cell 4, 298-309

7 Sonneborn A, Bockmühl DP, Gerads M, Kurpanek K, Sanglard D, Ernst JF. Protein kinase A encoded by TPK2 regulates dimorphism of Candida albicans. Mol Microbiol. 2000 Jan;35(2):386-96. doi: 10.1046/j.1365-2958.2000.01705.x. PMID: 10652099.

8 Lo HJ, Köhler JR, DiDomenico B, Loebenberg D, Cacciapuoti A and Fink GR. Nonfilamentous *C. albicans* mutants are avirulent. Cell. 1997 Sept;90(5)

9 Su, C, Lu, Y and Liu H Reduced TOR signalling sustains hyphal development in *Candida albicans* by lowering Hog1 basal activity. Mol Biol Cell. 2013 Feb; 24(3): 385-397.

10 Wang A, Raniga PP, Lane S, Lu Y and Liu H. Hyphal chain formation in Candida albicans: Cdc28-Hgc1 phosphorylation of Efg1 represses cell separation genes. Mol and Cell Biol. 2009 Aug; 29(16).

| Primer  Table S2. Primers and oligonucleotides used in this study | Sequence | Purpose and feature |
| --- | --- | --- |
| 1 | 5’agatctacgctaggatcgatatcaagcttatcgatATGAGTCATTTGGTACTG | pBA1-SFL1 junction |
| 2 | 5’GCATTCAATGTCTTgtcGCTTCTTCGTTTTATATTC | pBA1-SFL1S225D-GFP |
| 3 | 5’cacctttagacatatgcatgctTTCTAATTTTCTCTTTTTATGATCG | SFL1-SphI-yeGFP junction |
| 4 | 5’CGAAGAAGCgacAAGACATTGAATGCAC | pBA1-SFL1S225D-GFP |
| 5 | 5’CATTCAATGTCTTcgcGCTTCTTCGTTTTATATTC | pBA1-SFL1S225A-GFP |
| 6 | 5’CGAAGAAGCgcgAAGACATTGaatgcac | pBA1-SFL1S225A-GFP |
| 7 | 5’CTTATATGGTGGgatCCAAGTTTGGATTCATTTTATGTAgacCCAGGAGAAGAGTTTTC | pBA1-SFL1S137D, T146D-GFP |
| 8 | 5’CTTCTCCTGGgtcTACATAAAATGAATCCAAACTTGGatcCCACCATATAAGATGGG | pBA1-SFL1S137D, T146D-GFP |
| 9 | 5’CTTCTCCTGGcgcTACATAAAATGAATCCAAACTTGGagcCCACCATATAAGATGGG | pBA1-*SFL1S137A*, *T146A*-GFP |
| 10 | 5’CTTATATGGTGGgctCCAAGTTTGGATTCATTTTATGTAgcgCCAGG | pBA1-*SFL1S137A*, *T146A*-GFP |
| 11 | 5’cttcacctttagacatatgcatgcttttattcatattatcagtatcatcatc | pBA1-*SFL2S133D/A*, *S134D/A*-GFP |
| 12 | 5’caaAGATCTACGCTAGGATCGATATCAAGCTTATCGATatgagtaagaaaaatcctgg | pBA1-*SFL2S133D*/*A*, *S134D*/*A*-GFP |
| 13 | 5’GAGAAGATCAgacgatAACCATACTTCGGGAAG | pBA1-*SFL2S133D*, *S134D*-GFP |
| 14 | 5’GTATGGTTatcgtcTGATCTTCTCTTGATGTATACC | pBA1-*SFL2S133D*, *S134D*-GFP |
| 15 | 5’GAGAAGATCAgccgccAACCATACTTCGGGAAG | pBA1-*SFL2S133A*, *S134A*-GFP |
| 16 | 5’GTATGGTTggcggcTGATCTTCTCTTGATGTATACCAATG | pBA1-*SFL2S133A*, *S134A*-GFP |
| 17 | 5’ GCATTCAATGTCTTgtcGCTTCTTCGTTTTATATTC | pBA1-*SFL1S225D*-GFP |
| 18 | 5’ CGAAGAAGCgacAAGACATTGAATGCAC | pBA1-*SFL1S225D*-GFP |
| 19 | 5’ CATTCAATGTCTTcgcGCTTCTTCGTTTTATATTC | pBA1-*SFL1S225A*-GFP |
| 20 | 5’ CGAAGAAGCgcgAAGACATTGaatgcac | pBA1-*SFL1S225A*-GFP |
| 21 | 5’GATACTGATAATATGAATAAAAGCATGCATATGTCTAAAGGTGAAGAATTATTC | pBA1-*SFL2*-GFP |
| 22 | 5’GCTCAATTAACCCTCACTAAAGGGAACAAAAGCTGGGTACCTGCAGTTATTTGTACAATTCATCCATACC | pBA1-*SFL2*-GFP |
| 23 | 5’ TGGATTAGCTCGAGCATTTG | *CDC28* qRT-PCR |
| 24 | 5’ CCAACAGACCACATATCTACCC | *CDC28* qRT-PCR |
| 25 | 5’ CCAACTCCCTTGCTTTGTCG | *NRG1* qRT-PCR |
| 26 | 5’ GTTGGCCATGGACATTGGTG | *NRG1* qRT-PCR |
| 27 | 5’ TCTTACCTCAATCAGCATTA | *UME6* qRT-PCR |
| 28 | 5’ CAGCACTAACACTGACACC | *UME6* qRT-PCR |
| 29 | 5’ CCTATTCCAACAACTACAAT | *ALS3* qRT-PCR |
| 30 | 5’ TATTGAGTCAGTTGGATTAG | *ALS3* qRT-PCR |
| 31 | 5’ GTTGCTGGTGGTGGTAATGG | *YWP1* qRT-PCR |
| 32 | 5’ GCTGAATTGTCTGCCTTGGT | *YWP1* qRT-PCR |
| 33 | 5’ TTTTTCAACAAAAGCTGTTGCG | *ARG4* genomic |
| 34 | 5’ TCAAAAGACTCTCATTAGAGC | *ARG4* genomic |
| 35 | 5’GGAACAACTACCACAAACAATAACAACGTTAGTAATAACAATTCAACGGGGAAAACGACATTGAATGCACAAAAGGAAGTTGTCAATATAAAATCTTTACCACCGAC | dDNA CRISPR-CAS9  (double stranded gene fragment) |
| 36 | 5’CGTAAACTATTTTTAATTTGCGATATCCCATCTTATATGGGTTTTAGAGCTAGAAATAGC | Annealed for gRNA |
| 37 | 5’CGTAAACTATTTTTAATTTGCCATATAAGATGGGATATCGGTTTTAGAGCTAGAAATAGC | Annealed for gRNA |
| AHO1098 | 5’ CAAATTAAAAATAGTTTACGCAAG | prepare “entry vector” [1] |
| AHO1099 | 5’ GTTTTAGAGCTAGAAATAGCAAGTT | prepare “entry vector” [1] |
| 38 | 5’CGGGATCCCATGTCAACGTATTCTATACCCTATTAC | pEFG1-EFG1^T106A, T107A, T108A^-MYC |
| 39 | 5’CCACCAGGAATCaggcctCGAGTAGCAGCTGCCATGTGGGAAGATGAAAAAACTTTG | pEFG1-EFG1^T106A, T107A, T108A^-MYC |
| 40 | 5’GGCGACGCGTCGCTTTTCTTCTTTGGCAACAGTGCT | pEFG1-EFG1^T106A, T107A, T108A^-MYC |
| 41 | 5’CAATTCCAACCACCAGGAATCaggcctCGAGTAGCAGCTGCCATGTGGGAAGATG | pEFG1-EFG1^T106A, T107A, T108A^-MYC |
| 42 | 5’CAGGTGATGTtgctgcTTGtgcTTGTGGTGGTGGTGGTGGTAGTGGTG | pBA1-*SFL2^CN^*-GFP |
| 43 | 5’CTACCACCACCACCACCACAAgcaCAAgcagcaACATCACCTGGAATTC | pBA1-*SFL2^CN^*-GFP |
| 44 | 5’CCTTCAGAagcagcCCTcgcCAATGGggcagcCATcgcAGACGGATTTCTTGGCTTTGAATTGG | pBA1-*SFL2^CN^*-GFP |
| 45 | 5’CCAAGAAATCCGTCTgcgATGgctgccCCATTGgcgAGGgctgctTCTGAAGGTTCACC | pBA1-*SFL2^CN^*-GFP |
| 46 | 5’GTGCTTATTTTAGGtgcTGTagcGACATCTTTTGATAAATTATTACC | pBA1-*SFL2^CN^*-GFP |
| 47 | 5’CAAAAGATGTCgctACAgcaCCTAAAATAAGCACAAATTC | pBA1-*SFL2^CN^*-GFP |
| 48 | 5’GACTATGACTAGGTGCCAATGCTGACGTCACCGATAC | pBA1-*SFL2^CN^*-GFP |
| 49 | 5’GGTGACGTCAGCATTGGCACCTAGTCATAGTCAAAGTC | pBA1-*SFL2^CN^*-GFP |

1 Nguyen N QM, Hernday AD. An Efficient, Rapid, and Recyclable System for CRISPR-Mediated Genome Editing in Candida albicans. mSphere. 2017;2(2).

Restriction sites are underlined.

| #  Table S3. Plasmids used in this study | Plasmid | Description | Source |
| --- | --- | --- | --- |
| HLp549 | pBES116 | *C. albicans URA3* vector, integration at *ADE2* | [2] |
| HLp596 | pBA1 | *C. albicans ADH1* promoter in pBES116 | [1] |
| HLp471 | yEGFP3 | yeast-enhanced GFP for *C.albicans* | [3] |
| HLp1279 | pBA1-SFL2-GFP | Ca*SFL2-*SphI*-yEGFP3* in pBA1 | This study |
| HLp1280 | pBA1-SFL1-GFP | Ca*SFL1-*SphI*-yEGFP3* in pBA1 | This study |
| HLp1281 | pBA1-SFL1^S225D^-GFP | Ca*SFL1* *^S225D^-*SphI*-yEGFP3* in pBA1 | This study |
| HLp1282 | pBA1-SFL1S225A-GFP | Ca*SFL1* *^S225A^-*SphI*-yEGFP3* in pBA1 | This study |
| HLp1283 | pBA1-SFL1^S137D, T146D^-GFP | Ca*SFL1^S137D, T146D^-*SphI*-yEGFP3* in pBA1 | This study |
| HLp1284 | pBA1-SFL1^S137A, T146A^-GFP | Ca*SFL1^S137A, T146A^-*SphI*-yEGFP3* in pBA1 | This study |
| HLp1285 | pBA1-*SFL2^S133D, S134D^-GFP* | Ca*SFL2^S133D, S134D^-*SphI*-yEGFP3* in pBA1 | This study |
| HLp1286 | pBA1-*SFL2^S133A, S134A^-GFP* | Ca*SFL2^S133A, S134A^-*SphI*-yEGFP3* in pBA1 | This study |
| HLp1287 | pBA1-*SFL1^S137A^*^,^ *^T146A^*^,^ *^S225D^*-GFP | Ca*SFL1^S137A, T146A^*^,^ *^S225D^-*SphI*-yEGFP3* in pBA1 | This study |
| HLp1288 | pBA1-SFL1^S137A, T146A, S225A^-GFP | Ca*SFL1^S137A, T146A^*^,^ *^S225A^-*SphI*-yEGFP3* in pBA1 | This study |
| HLp1289 | pBA1-SFL1^S137D, T146D, S225D^-GFP | Ca*SFL1^S137D, T146D^*^,^ *^S225D^-*SphI*-yEGFP3* in pBA1 | This study |
| HLp1290 | pBA1-SFL1^S137D, T146D, S225A^-GFP | Ca*SFL1^S137D, T146D^*^,^ *^S225A^-*SphI*-yEGFP3* in pBA1 | This study |
| HLp1291 | gRNA_sfl1HSD | gRNA for *sfl1 ^DBD∆^* cloned into "entry vector" | This study |
| HLp1292 | pBA1-SFL2^CN^-GFP | Ca*SFL2^CN^-*SphI*-yEGFP3* in pBA1 | This study |
| HLp1262 | *C. albicans* HIS1 FLP | CAS9 plasmid pADH99 | [4] |
| HLp1263 | *C. albicans* gRNA | gRNA plasmid pADH100 | [4] |
| HLp695 | pACT1-EFG1-MYC |  | [5] |
| HLp893 | pACT1-EFG1^T206A, T207A, T208A^-MYC |  | [5] |

1. Cao, F., S. Lane, P. P. Raniga, Y. Lu, Z. Zhou, K. Ramon, J. Chen, and H. Liu. 2006. The Flo8 transcription factor is essential for hyphal development and virulence in *Candida albicans*. Mol. Biol. Cell 17:295-307.
2. Feng, Q., E. Summers, B. Guo, and G. Fink. 1999. Ras signaling is required for serum-induced hyphal differentiation in *Candida albicans*. J. Bacteriol. 181:6339-6346.
3. Cormack BP, Bertram G, Egerton M, Gow NAR, Falkow S, Brown AJP. Yeast-enhanced green fluorescent protein (yEGFP): a reporter of gene expression in Candida albicans. Microbiology (Reading). 1997 Feb;143 ( Pt 2):303-311. doi: 10.1099/00221287-143-2-303. PMID: 9043107.
4. Nguyen N QM, Hernday AD. An Efficient, Rapid, and Recyclable System for CRISPR-Mediated Genome Editing in Candida albicans. mSphere. 2017;2(2).
5. Wang A RP, Lane S, Lu Y, Liu H. Hyphal chain formation in *Candida albicans*: Cdc28-Hgc1 phosphorylation of Efg1 represses cell separation genes. Molecular and cellular biology. 2009;29(16).
