# Supplementary material for: Regulation of hyphal development by protein kinase A, stress-responsive MAP kinases, and calcineurin via transcription factors Sfl1 and Sfl2 in Candida albicans
Source: mSphere. 2026 Jan 8;11(2):e00689-25. doi: 10.1128/msphere.00689-25 (PMC12931275; doi:10.1128/msphere.00689-25)
Supplement: Fig. S1 and S2 — Sfl1 DBD structure and morphology of SFL2 PKA and calcineurin-binding mutants. [file msphere.00689-25-s0002.docx]

**
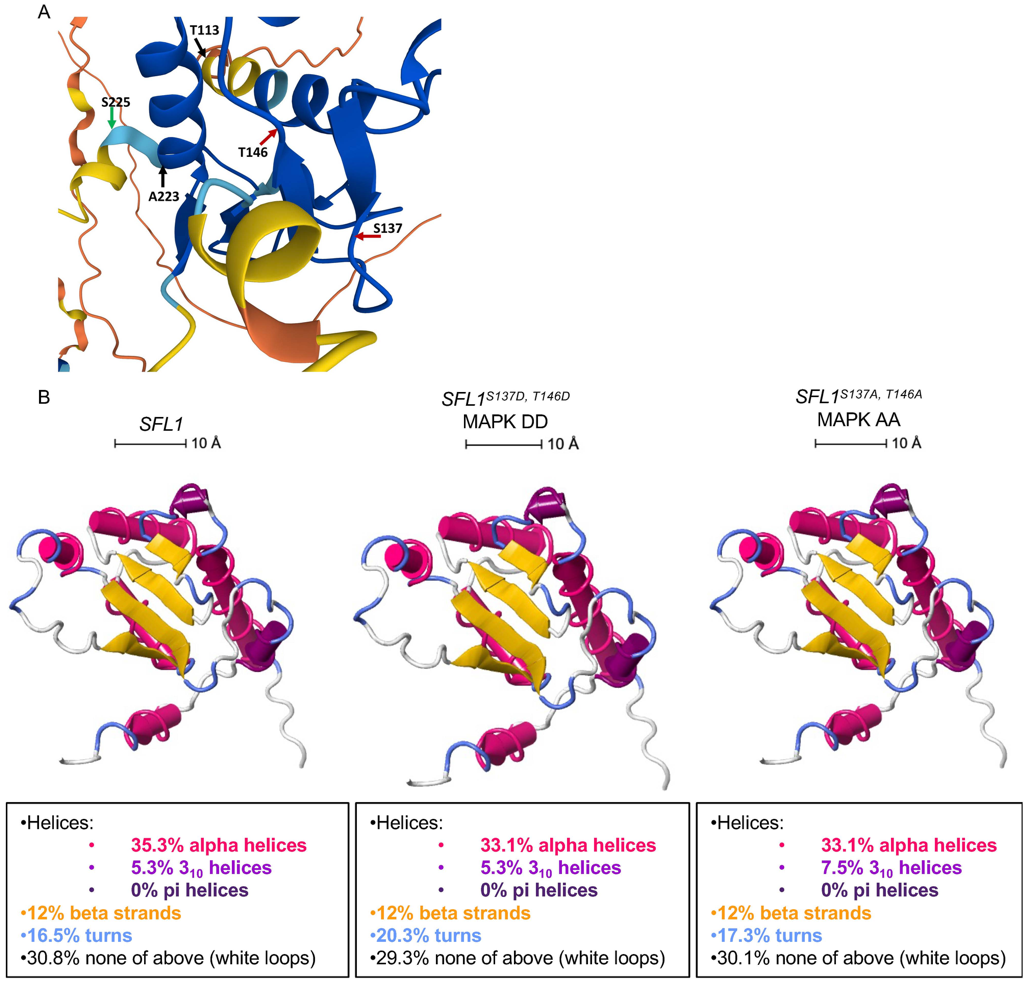
**

**Supplementary Fig 1.** **Alphafold structure of Sfl1 DNA binding domain and predicted PKA and MAPK phosphorylation sites.** (A) Ribbon diagram of Sfl1 DBD domain. Black arrows indicate start and end of DBD. Green arrow indicates PKA phosphorylation site. Red arrows indicate predicted MAPK phosphorylation sites. Nucleotides modeled include DBD with neighboring serine (T113-S225) plus 10 additional residues on each side. (B) Comparison of secondary structure of Sfl1 DBD domain and Sfl1 DBD with mutations in predicted MAPK phosphorylation sites.


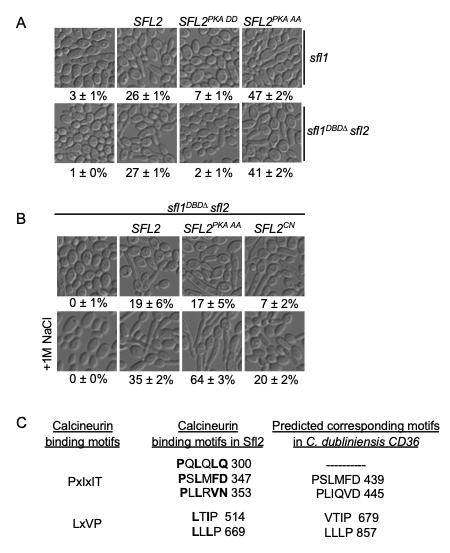


**Supplementary Fig 2.** **Morphology of *SFL2* PKA and calcineurin-binding mutations*.***

(A) Morphology of indicated strains grown overnight in YPD medium at 30°C. (B) Morphology of indicated strains grown overnight culture in YPD at 30°C, with or without NaCl. Images are representative of two biological replicates. (C) Predicted calcineurin binding motifs in *C. dubliniensis CD36,* corresponding to *C. albicans SFL2*.
